# PRSS3/mesotrypsin as a putative regulator of the biophysical characteristics of epidermal keratinocytes in superficial layers

**DOI:** 10.1038/s41598-024-63271-w

**Published:** 2024-05-29

**Authors:** Moeko Kida, Junya Abe, Haruna Hori, Yohei Hirai

**Affiliations:** Department of Biomedical Sciences, Graduate School of Science and Technology, Kwansei Gakuin University, 1 Gakuen-Uegahara, Sanda, 669-1330 Japan

**Keywords:** Epidermis, PRSS3/mesotrypsin, Cell shape, Growth arrest, Tight junction, Keratinocyte, Cell adhesion, Cell growth, Cell polarity, Cell biology, Molecular biology

## Abstract

Mesotrypsin, encoded by the PRSS3 gene, is a distinctive trypsin isoform renowned for its exceptional resistance to traditional trypsin inhibitors and unique substrate specificity. Within the skin epidermis, this protein primarily expresses in the upper layers of the stratified epidermis and plays a crucial role in processing pro-filaggrin (Pro-FLG). Although prior studies have partially elucidated its functions using primary cultured keratinocytes, challenges persist due to these cells' differentiation-activated cell death program. In the present study, HaCaT keratinocytes, characterized by minimal endogenous mesotrypsin expression and sustained proliferation in differentiated states, were utilized to further scrutinize the function of mesotrypsin. Despite the ready degradation of the intact form of active mesotrypsin in these cells, fusion with Venus, flanked by a peptide linker, enables evasion from the protein elimination machinery, thus facilitating activation of the Pro-FLG processing system. Inducing Venus-mesotrypsin expression in the cells resulted in a flattened phenotype and reduced proliferative capacity. Moreover, these cells displayed altered F-actin assembly, enhanced E-cadherin adhesive activity, and facilitated tight junction formation without overtly influencing epidermal differentiation. These findings underscore mesotrypsin's potentially pivotal role in shaping the characteristic cellular morphology of upper epidermal layers.

## Introduction

The epidermis, a stratified epithelium comprising basal, spinous, granular, and cornified layers, is pivotal for preserving skin integrity. Originating in the basal layer adjacent to the basal membrane, nascent keratinocytes undergo growth arrest and sequential ascent through these layers, during which they undergo differentiation and stratification. After reaching the granular layer, cells undergo significant morphological changes, adopting a flattened morphology, and modifying intercellular adhesive structures^1–4^. Subsequently, cells modulate the expression and activity of several molecular elements akin to quiescent or senescent cells^5,6^, and cells ultimately undergo enucleation and desquamation at the outermost cornified layer^2,3^. While epidermal terminal differentiation is concomitant with substantial changes in lipid synthesis and plasma membrane composition, these morphological changes might be caused by dynamic changes in the expression, distribution, and composition of the cytoskeleton and intercellular junctional apparatus^2,3,7^. For instance, keratin intermediate filaments, which are crucial for maintaining physical properties and stabilizing desmosomes connecting sub- and interlaminar borders to maintain tissue structure^7,8^, undergo dramatic changes in type and abundance during stratification^8,9^. Furthermore, actin microfilaments regulate not only cell shape but also adherens junctions (AJs), maintaining specific adhesion within each layer across the entire epidermis^4,10^, and tight junctions (TJs), contributing to barrier function in the upper granular layer^9,11^.

Filaggrin (FLG) has emerged as one of the key regulators of cytoskeletal dynamics, particularly during the later stages of epidermal stratification^1^. Initially, expressed as a large phosphorylated precursor, pro-filaggrin (Pro-FLG), in the granular layers exists as nonfunctional keratohyalin granules. Pro-FLG, comprising an N-terminal calcium-binding domain (FLG-N) and multiple filaggrin units (FLG), undergoes rapid processing and release by various proteases, including kallikrein5 (KLK5), KLK7, calpain, RACE4, SASPase, and mesotrypsin^12–14^. FLG-N activates the cell death program in granular cell nuclei, while FLG not only bundles keratin intermediate filaments but also influences actin dynamics, controlling cell shape and cell–cell adhesion^2,12,15,16^. Additionally, FLG acts as a component of cornified cell envelopes, robust structures formed beneath the cell membrane^15,17^. Among the molecular elements responsible for processing Pro-FLG, serine proteases, including KLKs and mesotrypsin (also known as trypsin3 or PRSS3 protein), have emerged as potential regulators of the morphological characteristics of the upper layer epidermis given their exclusive expression and activation at the granular and cornified layers^18,19^.

While epidermal differentiation and stratification are spatiotemporally regulated by several proteases^20^, we specifically directed our attention towards mesotrypsin due to its pivotal role in Pro-FLG processing. This protease represents an atypical trypsin isoform that displays peculiar resistance to common trypsin inhibitors and exhibits substrate specificity^21,22^. In the epidermis, enterokinase, or cathepsin B activates this protease through cleavage at a DDDDK-I site^23–25^, facilitating its proteolytic function in the cytoplasm of the granular layer and the extracellular space of the cornified layer^18,23^. Crucially, in addition to Pro-FLG processing, mesotrypsin activates KLK5, another pro-FLG processing enzyme, and degrades the KLK inhibitor LEKT1^18^, leading to the degradation of corneodesmosomes, the primary desmosomes in cornified layers^18,19^. While epidermal mesotrypsin has been known for more than a decade, earlier studies have primarily highlighted its effects on the activation of the cell death program via FLG-N production^14^ and desquamation via KLK5/LEKT1 system activation^18^. This difference may be attributed to its plausible function in terminally differentiated, dying keratinocytes.

HaCaT keratinocytes exhibit differentiation potential comparable to that of normal human epidermal keratinocyte (NHEK) cells and serve as reliable keratinocyte model cells^26–28^. However, unlike normal keratinocytes, HaCaT cells can proliferate in differentiated states without manifesting a flattened cell morphology^26,28^. Recent observations indicate that these cells maintained in conventional high-calcium medium express significant amounts of Pro-FLG irrespective of differentiation state and exhibit insufficient Pro-FLG processing systems^12^. Building upon these results, we sought to elucidate the role of mesotrypsin in keratinocytes utilizing HaCaT cells harboring a tetracycline (tet)/doxycycline (dox)-inducible expression cassette for active mesotrypsin. Although mesotrypsin appears susceptible to rapid degradation by the proteasomal protein elimination system in these cells, mesotrypsin fused with modified GFP (Venus) demonstrated resilience to such degradation. The recombinant form of the GFP-mesotrypsin fusion protein degraded Pro-FLG extracted from HaCaT cells. A comparison of cells expressing Venus-mesotrypsin and Venus suggested that mesotrypsin might play a crucial role in shaping the morphological characteristics of upper-layered keratinocytes but not in differentiation.

## Results

### Expression of Pro-filaggrin and mesotrypsin in NHEK and HaCaT cells

**Figure 1:**
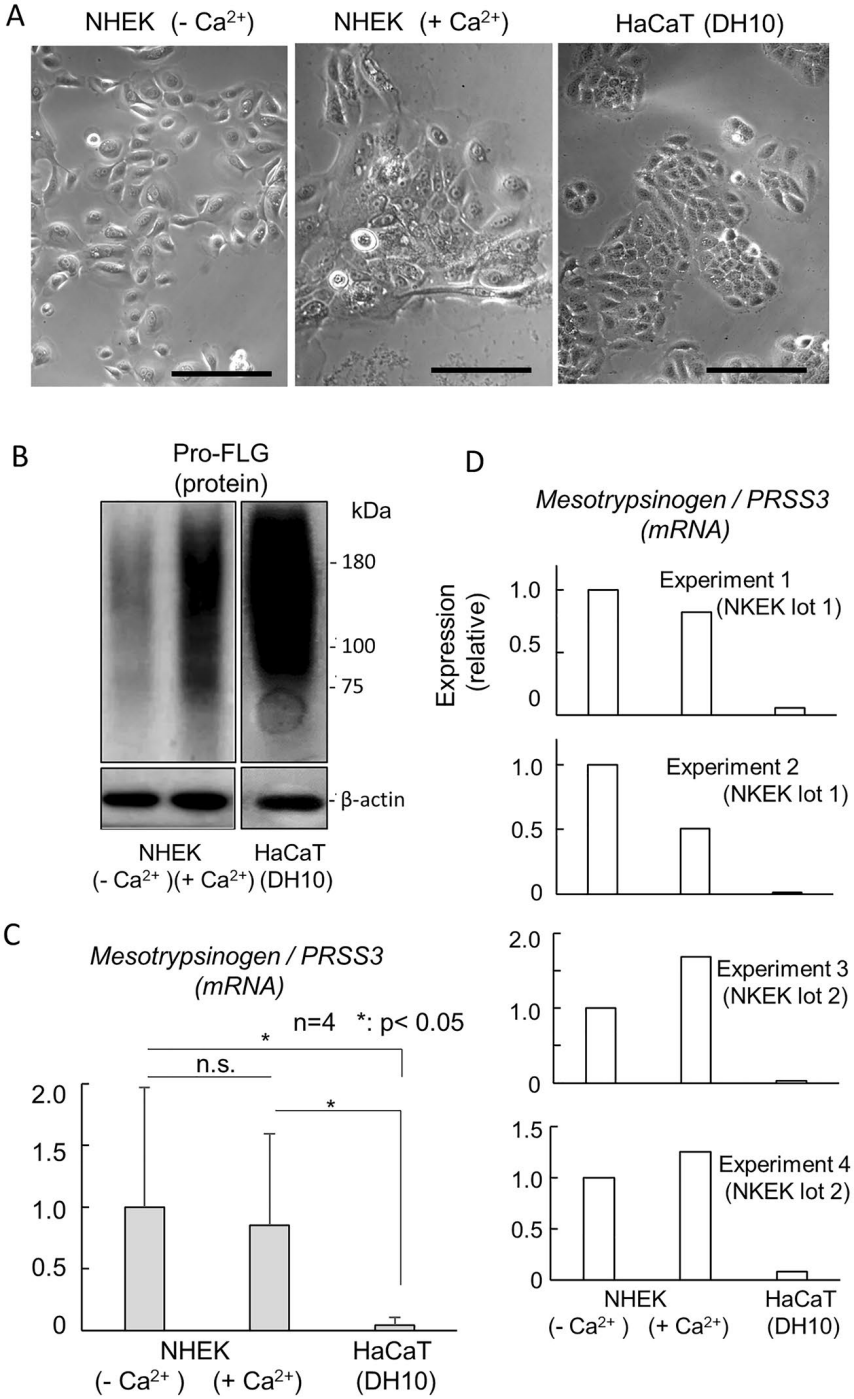
Expression of Pro-FLG and its processing protease mesotrypsin in NHEK and HaCaT cells. (**A**) Morphological characterization of model cells. NHEK cells were treated with (+ Ca^2+^) or without (− Ca^2+^) 2 mM CaCl_2_ for 2 days. Right, HaCaT cells cultured in DH10 medium. NHEK cells subjected to Ca^2+^ treatment undergo differentiation and exhibit a flattened morphology. Scale bars, 200 µm. (**B**) Expression of Pro-FLG and its processing enzyme mesotrypsin. The Pro-FLG protein is abundantly expressed in both differentiated NHEK and HaCaT cells. (**C**) Analysis of the mRNA expression of the mesotrypsinogen gene (*PRSS3*) revealed that HaCaT cells demonstrate the considerably lower transcriptional activity of the mesotrypsin gene compared to NHEK cells. n = 4, *p < 0.05. There are significant variations in error bars among NHEK cell samples, attributed to substantial differences in the basal expression of *PRSS3* in NHEK (lot 1) and NHEK (lot 2). (**D**) The expression of *PRSS3* was evaluated in each experiment, elucidating that its upregulation associated with differentiation was exclusively observed in NHEK (lot 2).

In the skin, pro-filaggrin and its processing enzyme, mesotrypsin, are almost exclusively detected within nonmitotic and differentiated keratinocytes situated in the epidermal granular and cornified layers^18,25^. Within NHEK cells, while Pro-FLG is minimally expressed even in undifferentiated states, as previously demonstrated^29^, this protein undergoes a significant upregulation upon the induction of differentiation facilitated by CaCl_2_. This induction prompts cells to exit the cell cycle^11^, accompanied by a flattened morphology (Fig. 1A,B). In contrast, HaCaT cells exhibit abundant expression of Pro-FLG and maintain proliferative capacity as differentiated keratinocytes in a standard high-calcium medium, without altering their morphology or proliferation potential (Fig. 1A,B). Conversely, mesotrypsinogen, the precursor of mesotrypsin, is consistently detected in NHEK cells, irrespective of their differentiation status, as reported previously^23^, However, this study unveils minimal expression of mesotrypsinogen/mesotrypsin in HaCaT cells (Fig. 1C). Notably, in NHEK cells, both the expression level and the differentiation-associated upregulation exhibit considerable lot-to-lot variation (Fig. 1D).

### Generation of HaCaT cell derivatives for functional analyses of mesotrypsin

**Figure 2:**
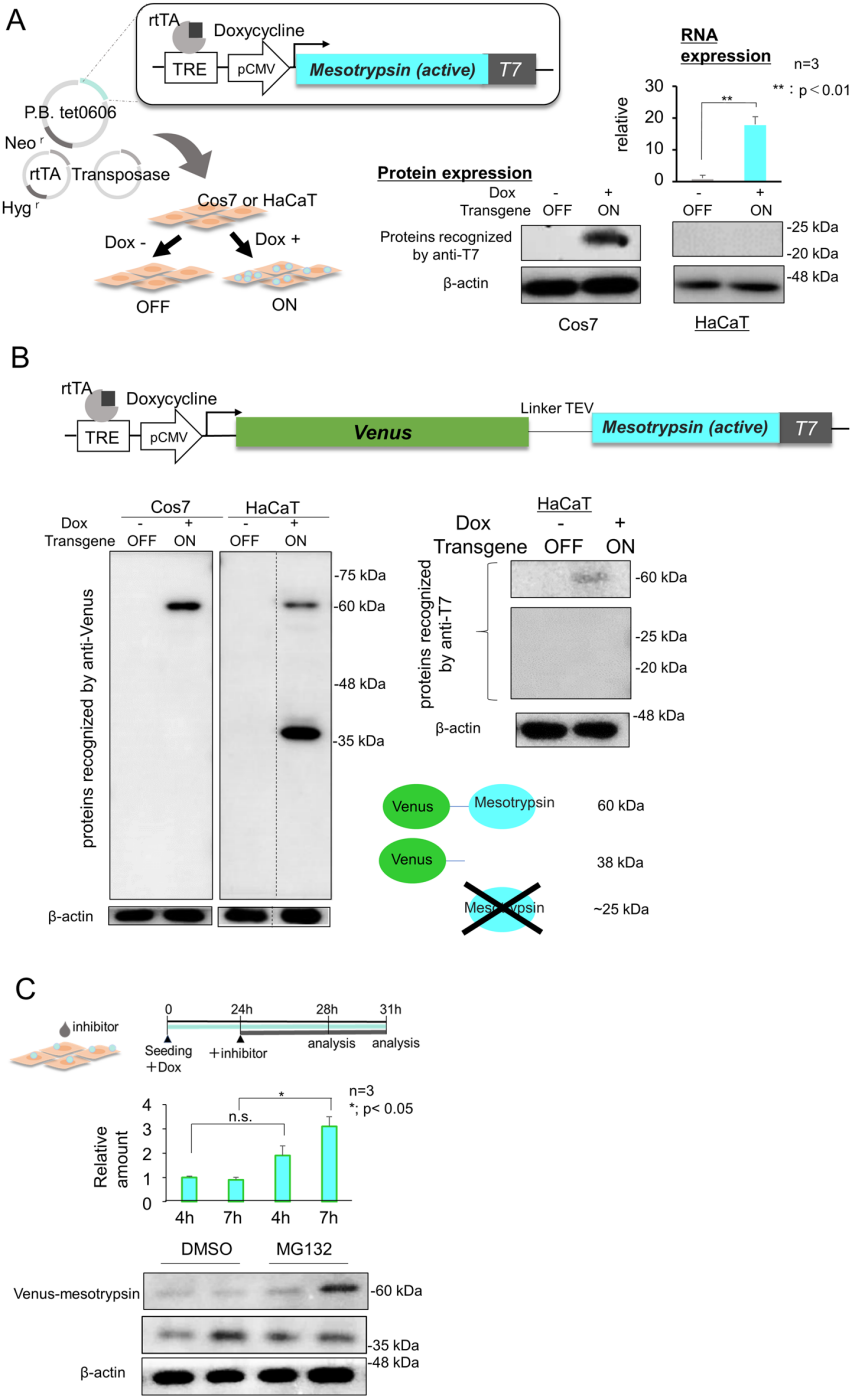
The regulation of mesotrypsin expression in HaCaT cells is governed by a protein elimination system. (**A**) Schematic representation delineating doxycycline-inducible expression constructs for active mesotrypsin (left) and subsequent analyses of the transgene product in Cos7 or HaCaT cells (right). TRE, tetracycline/doxycycline-responsive element. T7-tagged active mesotrypsin was successfully generated in Cos7 cells, while its production was notably absent in HaCaT cells, despite its mRNA being expressed. n = 3, **p < 0.01. (**B**) Upper, Presentation of the expression construct featuring a Venus-mesotrypsin fusion protein linked by a peptide linker and TEV recognition site. Lower, Assessment of the exogenous Venus-mesotrypsin fusion protein in both Cos7 and HaCaT cells. Cos7 cells exclusively express the fusion protein (~ 60 kDa), whereas HaCaT cells produce an additional 38 kDa protein, recognized by anti-Venus/GFP antibodies, following doxycycline treatment. Antibodies targeting the T7 tag associated with mesotrypsin were used to identify the 60 kDa fusion protein, but the protein was not reactive toward the ~ 25 kDa Venus-free mesotrypsin in HaCaT cells. (**C**) Treatment with the proteasome inhibitor MG132 effectively prevented the time-dependent reduction in the Venus-mesotrypsin fusion protein (60 kDa). n = 3, *p < 0.05.

**Figure 3:**
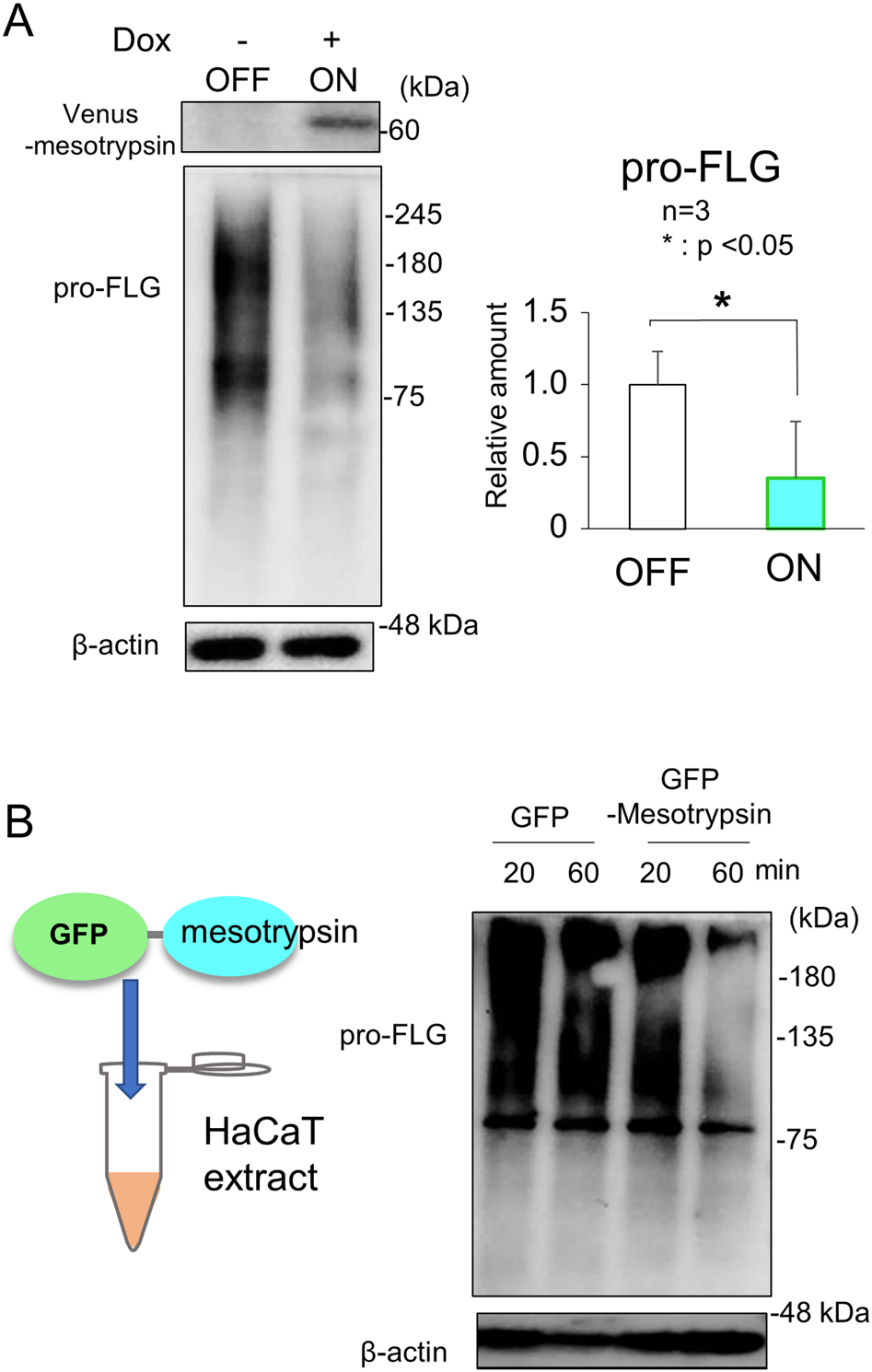
The Venus/GFP-mesotrypsin fusion protein exhibited pro-FLG-processing activity. (**A**) HaCaT cells expressing the Venus-mesotrypsin fusion protein exhibit a reduction in Pro-FLG levels. n = 3, *p < 0.05. (**B**) Recombinant mesotrypsin fused with GFP, an intrinsic derivative of Venus, degrades pro-FLG while leaving β-actin unaffected in the cellular extracts derived from HaCaT cells.

Expanding on the expression patterns of mesotrypsin and its representative substrate, Pro-FLG, we sought to elucidate the functional role of mesotrypsin in HaCaT cells. Initially, we engineered HaCaT cells harboring a tet/dox-inducible expression construct featuring active mesotrypsin tagged with a T7 peptide at the C-terminus. To achieve this, we employed the tetracycline-responsive element (TRE)-containing plasmid PBtet0606, housing the expression cassette for a neomycin resistance gene independent of tet/dox induction. Additionally, we utilized the reverse tet/dox transactivator (rtTA) plasmid containing a hygromycin resistance gene, along with a piggyBac (PB) transposase plasmid (Fig. 2A). While cells resistant to both antibiotics expressed mesotrypsin mRNA in response to dox treatment, the protein expression of mesotrypsin was detected only in Cos7 cells and not in HaCaT cells (Fig. 2A). This observation suggested that HaCaT cells exhibit either translational inhibition or a protein-eliminating system for active mesotrypsin. Subsequently, we evaluated the expression of the Venus-mesotrypsin fusion protein. The approximately 60 kDa protein was identified using an anti-GFP/Venus antibody in both Cos7 and HaCaT cells, indicating successful expression of the fusion protein. However, a distinct protein band at approximately 38 kDa was detected with the antibody against the N-terminal Venus but not with the C-terminal T7-tag. Additionally, T7-tagged proteins, except for the full-length ~ 60 kDa protein, were not discernible (Fig. 2B). This finding suggested that a significant portion of the mesotrypsin domain in the fusion protein undergoes rapid degradation in HaCaT cells. Treatment with the potent proteasome inhibitor MG132, as opposed to the control DMSO, impeded the degradation of mesotrypsin (Fig. 2C). This finding suggested that HaCaT cells possess posttranslational regulatory systems for the mesotrypsin protein, complementing its transcriptional suppression (Fig. 1B). We then investigated whether the Venus-mesotrypsin fusion protein retains its proteolytic activity. The Pro-FLG protein, a substrate of mesotrypsin processing, exhibited a reduction in abundance and a slight alteration in its molecular weight upon Venus-mesotrypsin expression (Fig. 3A). In addition, the recombinant form of the GFP-mesotrypsin fusion protein, but not GFP alone, degraded the Pro-FLG protein extracted from HaCaT cells, leaving a 76 kDa FLG dimer (Fig. 3B), indicating that the GFP/Venus-mesotrypsin fusion protein, which evades proteasomal degradation, partially exerts effects of mesotrypsin. Notably, however, while mesotrypsin is recognized for cleaving off the N-terminal domain from Pro-FLG exclusively, the amount of Pro-FLG fragments was dramatically decreased. This suggests that HaCaT cells harbor enzymes that facilitate further processing of these fragments. Indeed, we observed the expression of KLK5, a protease activated by mesotrypsin to further process Pro-FLG fragments into FLG, along with another FLG-processing protease, calpain-1 (Supplementary Fig. 1). Furthermore, it has been reported that FLG is very unstable in HaCaT cells, which express caspase14 to digest FLG into small peptides known as natural moisturizing factors (NMFs)^12^.

### Effect of mesotrypsin on HaCaT cell behaviors

**Figure 4:**
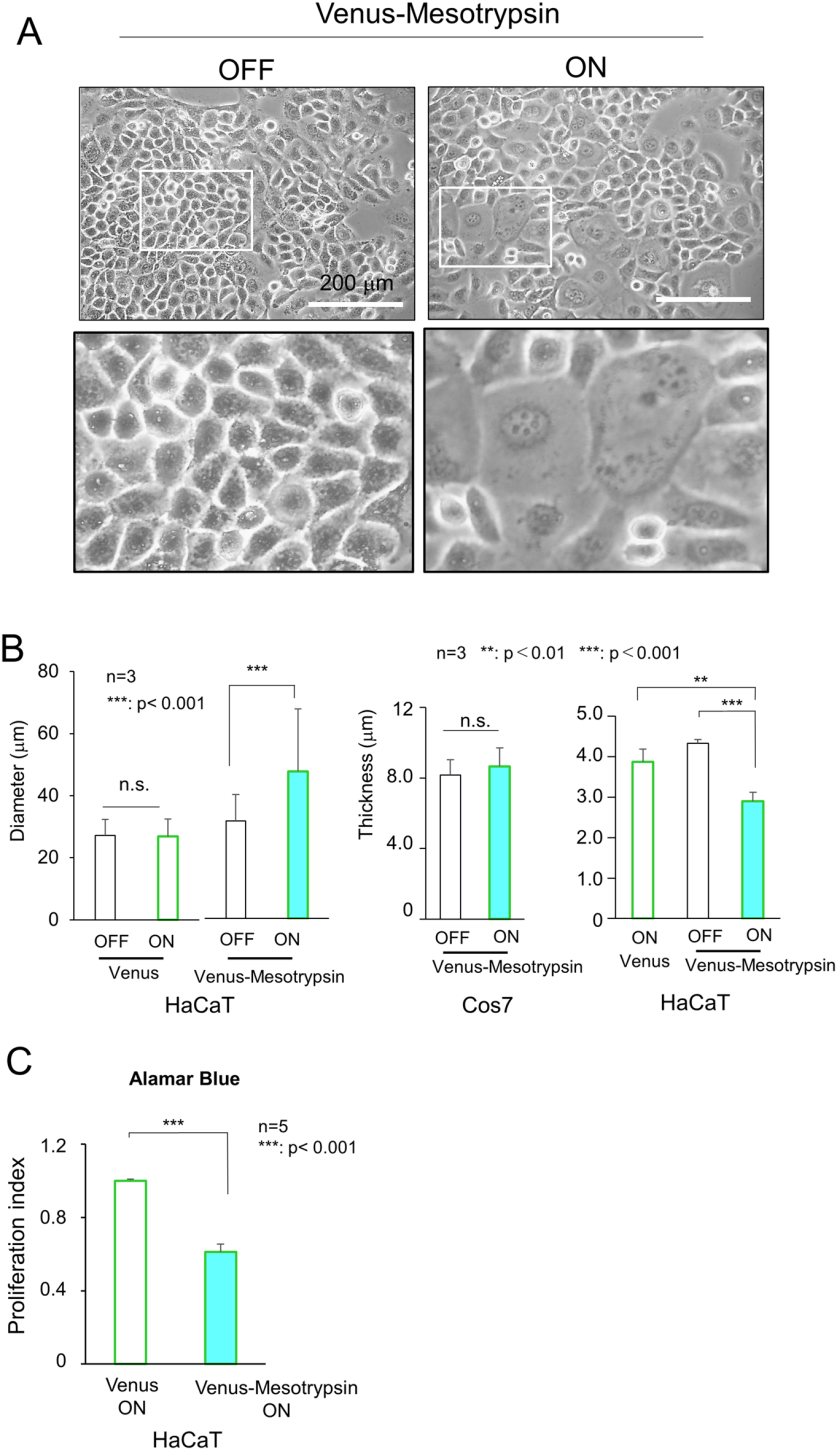
Mesotrypsin induces a flattened morphology and growth arrest in HaCaT cells. (**A**) Phenotypic depiction of HaCaT cells expressing Venus-mesotrypsin (right) and those without expression (left). Scale bars, 200 µm. (**B**) Left, Quantitative assessment of cell size. The expression of Venus-mesotrypsin, as opposed to Venus alone, results in a significant increase in cell size. Middle and right, Determination of cell thickness using z-stack images. n = 3, **p < 0.01. ***p < 0.001. Randomly selected 20 cells were analyzed in each experiment. In contrast to Cos7 cells, HaCaT cells exhibit a flattened morphology upon Venus-mesotrypsin expression. (**C**) The Alamar Blue assay revealed growth arrest in HaCaT cells expressing Venus-mesotrypsin. n = 5, ***p < 0.001.

Upon induction of Venus-mesotrypsin expression, HaCaT cells exhibited an increase in size, and z-stack image analysis revealed a pronounced manifestation of a flattened morphology, a phenomenon conspicuously absent in Cos7 cells (Fig. 4A,B and Supplementary Fig. S2). Additionally, the Alamar Blue assay illustrated that mesotrypsin induces a significant reduction in the proliferation of HaCaT cells (Fig. 4C). These morphological shifts, coupled with the growth arrest, epitomize hallmark features akin to keratinocytes residing in the granular and cornified layers, practically mirroring the characteristics of fully differentiated NHEK cells^32^. This finding suggested that mesotrypsin plays a pivotal role as a regulator of the morphological attributes distinctive to the differentiated upper layer of the epidermis.

### Effect of mesotrypsin on the formation of AJs and TJs

**Figure 5:**
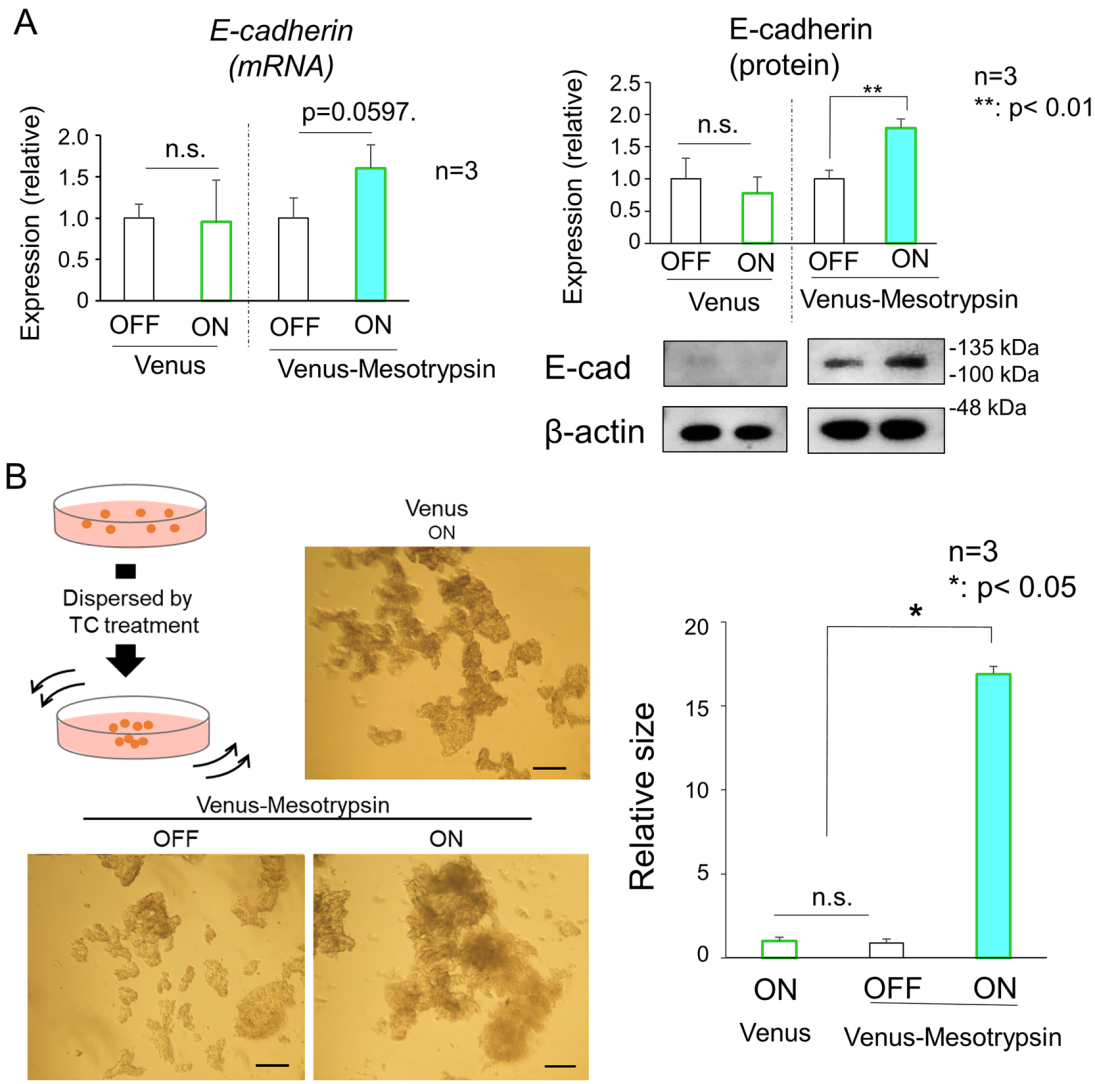
Mesotrypsin enhances adherens junctions (AJs). (**A**) Assessment of E-cadherin mRNA (left, n = 3) and protein (right, n = 3, **p < 0.01) expression. In addition, HaCaT cells expressing Venus-mesotrypsin exhibited upregulated E-cadherin. (**B**) Upper left, A schematic representation of the cell aggregation assay designed to evaluate cadherin activity. Cells subjected to trypsin treatment in the presence of calcium (TC treatment) eliminate most cell adhesion molecules, leaving cadherins intact. Disrupted cells in rotational culture reassembled into aggregates, the size of which reflects the activity of E-cadherin. Middle and lower, Representative images of reaggregated cells. Scale bars, 200 µm. Right, Quantification of aggregate sizes. n = 3, *p < 0.05. The expression of Venus-mesotrypsin, as opposed to Venus alone, results in the formation of large aggregates, indicating heightened cadherin activity.

**Figure 6:**
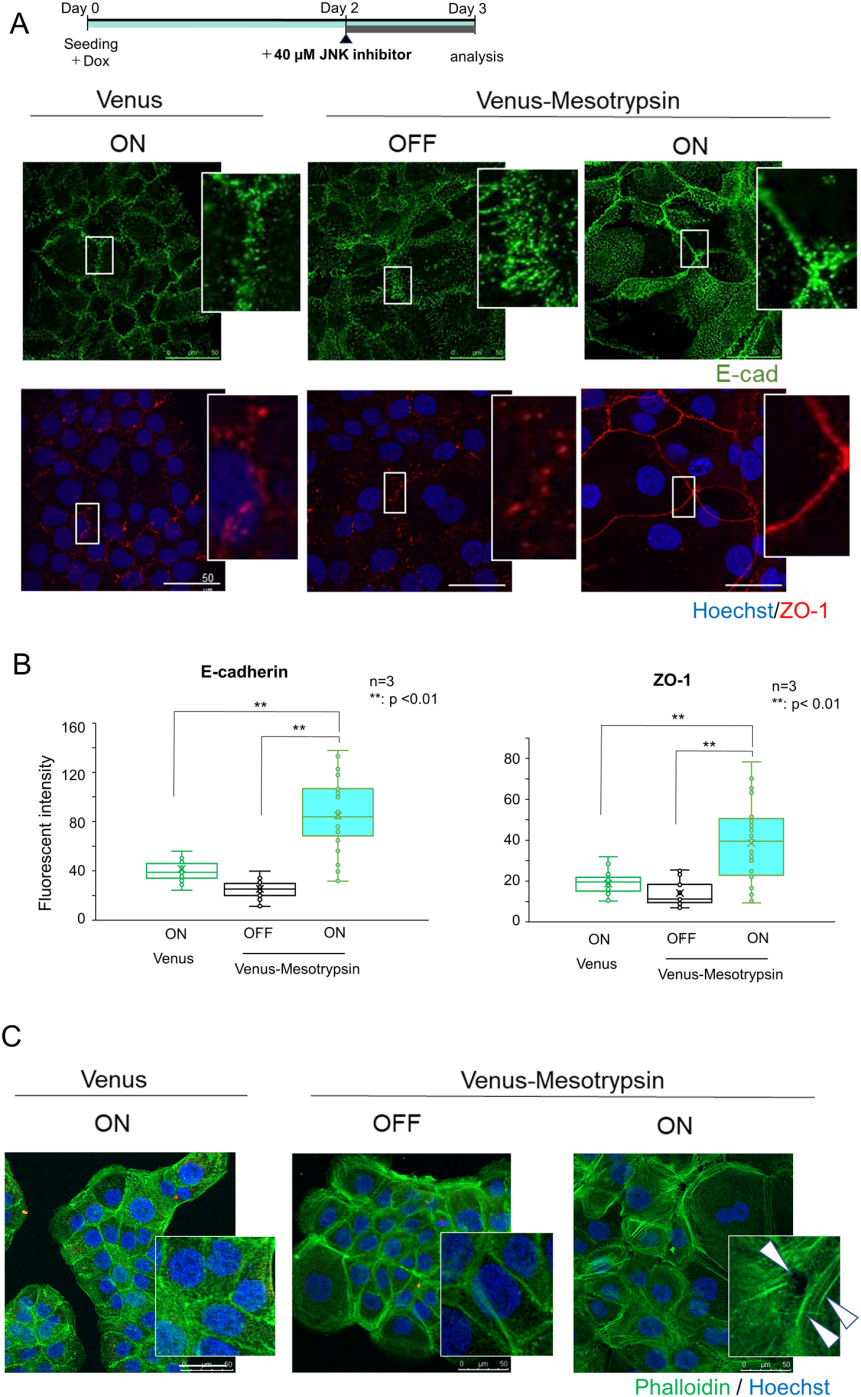
Mesotrypsin plays a supportive role in forming adherens junctions (AJs) and tight junctions (TJs). (**A**) The experimental timeline for the analysis of TJ formation is depicted at the top. Immunolocalization of E-cadherin (green) and ZO-1 (red) in HaCaT cells expressing Venus only (left) or Venus-mesotrypsin (right). In the middle, HaCaT cells without transgene expression were generated. The cells were counterstained with Hoechst 33258. Scale bars, 50 µm. (**B**) Quantification of the accumulation of E-cadherin (left) and ZO-1 (right) in HaCaT cells expressing Venus-mesotrypsin. The signal intensities of E-cadherin (left) and ZO-1 (right) per unit length of the cell-edge lines were measured. n = 3, **p < 0.01. Randomly selected 30 (left) or 29 cells (right) were analyzed in each experiment. The presence of Venus-mesotrypsin significantly accelerates the accumulation of E-cadherin and ZO-1 at cell–cell contact sites. Treatment with only the JNK inhibitor was insufficient for promoting TJ formation in HaCaT cells expressing Venus alone or lacking Venus-mesotrypsin.

Another distinctive feature of the granular layer is the formation of tight junctions (TJs), which are discernible intercellular adhesion complexes governing paracellular permeability and conferring barrier functions upon the epidermis^33,34^. Considering the prerequisite for stable adherens junctions (AJs) in TJ formation^35^, our initial investigation focused on the impact of mesotrypsin on the physical characteristics of AJs. Our findings indicate that Venus-mesotrypsin elevates the expression of E-cadherin, the primary constituent of epidermal AJs (Fig. 5A). To evaluate the strength of cadherin-mediated cell–cell adhesion, cells depleted of most cell surface proteins, except for cadherins (Supplementary Fig. S3), underwent mechanical dissociation and subsequent re-aggregation. These cells demonstrated heightened adhesive capabilities following the expression of Venus-mesotrypsin but not Venus alone (Fig. 5B), suggesting that mesotrypsin stabilizes or fortifies adherens junctions in HaCaT cells. Subsequently, examinations were conducted to assess the influence of mesotrypsin on TJ formation. The cytoplasmic translocation of ZO-1, the major scaffolding protein of TJs, is considered a representative hallmark of TJ formation in epidermal keratinocytes and is induced in HaCaT cells by simultaneous treatment with 10 mM calcium and a JNK inhibitor^35,36^. Our findings demonstrated that rapid ZO-1 translocation, along with E-cadherin accumulation at cell–cell contact sites, was induced in HaCaT cells expressing Venus-mesotrypsin even in the absence of elevated calcium concentrations, highlighting the facilitative role of mesotrypsin in TJ formation (Fig. 6A,B). Concurrent with the changes in the localization of critical components of the cell–cell adhesion apparatus, mesotrypsin significantly influenced the dynamics of F-actin, directing stress fiber assembly in HaCaT cells (Fig. 6C).

### Effect of mesotrypsin on growth and differentiation

**Figure 7:**
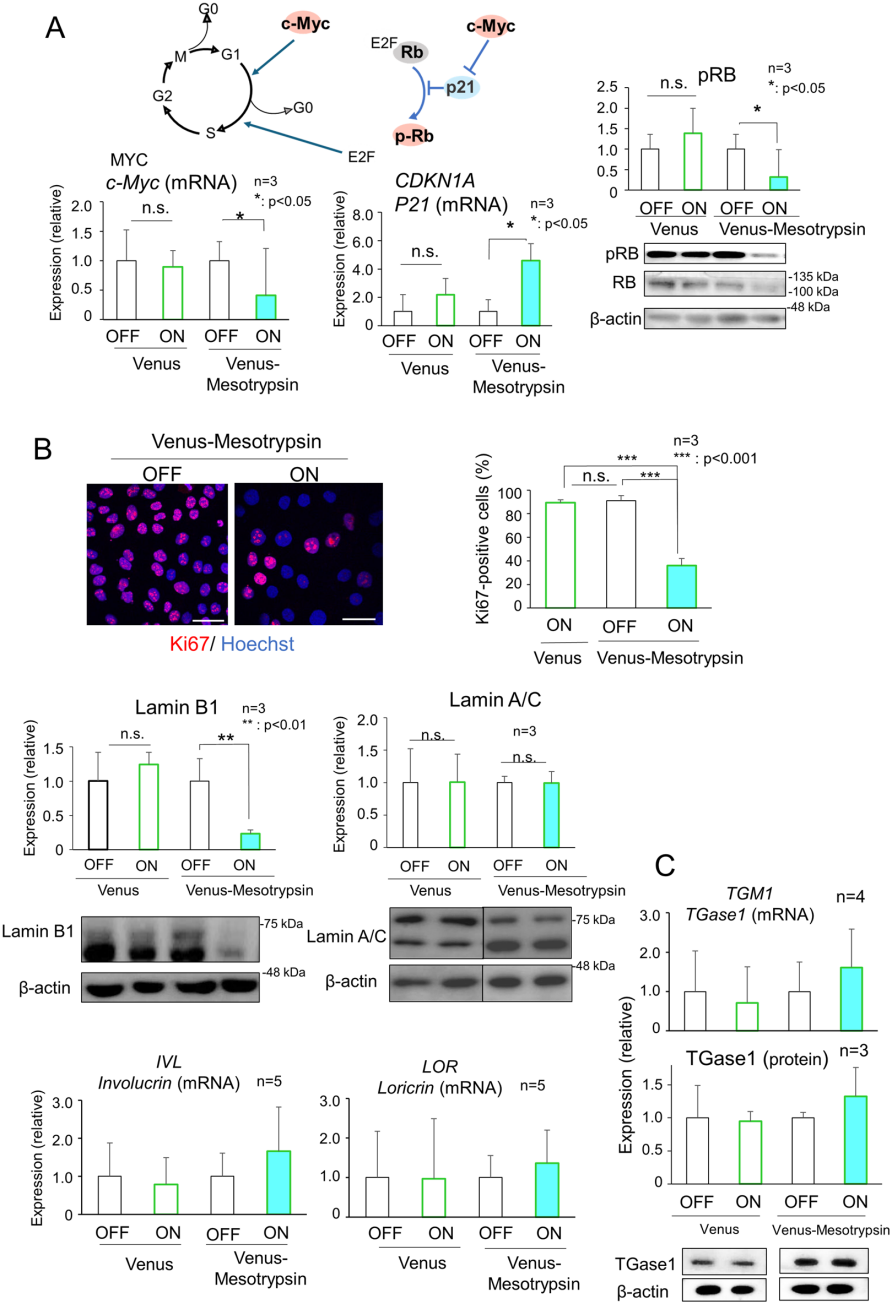
Mesotrypsin induces G_1_ growth arrest and cell cycle exit without impacting epidermal differentiation. (**A**) Expression profile of key elements involved in cell cycle progression. Venus-mesotrypsin, as opposed to Venus alone, leads to the downregulation of *MYC* (*c-Myc)* and upregulation of *CDKN1A (p21)* (left and middle). Concurrently, there was a reduction in RB phosphorylation. n = 3, *p < 0.05. (**B**) Upper, Immunostaining and quantification of the Ki67-positive cell population (depicted in red in the upper left panels, n = 3, ***p < 0.001.). The cells were counterstained with Hoechst 33258. Scale bars, 50 µm. Lower, when Lamin B1 was downregulated while lamin A/C was unaffected, the cell cycle was arrested in HaCaT cells expressing Venus-mesotrypsin. n = 3, **p < 0.01. (**C**) Expression analysis of various markers associated with epidermal differentiation, such as *IVL* (*involucrin*) (n = 5), *LOR* (*loricrin*) (n = 5), and TGase-1 (mRNA; n = 4, protein; n = 3), revealed no significant changes in HaCaT cells upon induction with Venus-mesotrypsin.

Given that mitotic keratinocytes are not detected within the upper-layer epidermis, we tested the growth profile of the mesotrypsin-expressing HaCaT cells. As mesotrypsin expression induced significant suppression of cell growth (Fig. 4C), we assessed the expression profiles of proteins regulating cell cycle progression, namely, Rb, c-Myc, and P21^37,38^. Rb governs the expression of genes essential for the transition from the G1 to S phase of the cell cycle, c-Myc orchestrates the transition from G0/G1 to S phase by activating the cyclin-dependent kinase (CDK) complex, and p21 serves as a cyclin-dependent kinase inhibitor that impedes positive feedback loops for G1 progression. Our findings revealed that cells expressing mesotrypsin exhibited a significant reduction in the level of inactive phosphorylated Rb, downregulation of c-Myc, and upregulation of P21, indicating growth arrest in the G1 phase (Fig. 7A). In addition to these effects, the number of Ki67-positive cells was significantly decreased (Fig. 7B), suggesting that mesotrypsin induced cell cycle exit. We detected downregulation of the senescence biomarker lamin B1 while leaving other cognate lamins, such as lamin A/C, unaffected (Fig. 7B). A decrease in the expression of laminB1 is also observed in the upper layers of the skin epidermis^5^. The induction of G1 arrest and cell cycle exit account for the observed effects on cell proliferation. In terms of differentiation, although HaCaT cells maintained for a long period in conventional high-calcium medium retain their differentiation potential^39,40^ and mesotrypsin diminishes the abundance of the differentiation marker Pro-FLG (Fig. 3), the expression of canonical differentiation markers, including TGase1, Loricrin, and Involucrin, remains unaffected (Fig. 7C). These findings suggest that mesotrypsin may play a pivotal role in inducing cell cycle exit and shaping the morphological characteristics of upper-layered keratinocytes without significantly impacting the differentiation process.

## Discussion

Epidermal keratinocytes exhibit distinct characteristics, specifically undergoing substantial alterations in cell morphology, physical attributes, membrane composition, and intercellular junctional complexes during the differentiation/stratification process^41,42^. To cause such profound changes, spatiotemporal regulation of the functional expression of numerous molecular components is imperative^43–45^. Notably, our focus centers on mesotrypsin, given its role as a serine protease involved in processing Pro-FLG to generate FLG-N, a process pivotal for epidermal apoptosis. Additionally, mesotrypsin activates KLKs to produce multiple FLG units, crucial for both aggregating keratin filaments and contributing to the formation of the cornified cell membrane in upper-layer keratinocytes. Moreover, most mesotrypsin isoforms found in keratinocytes lack the signal peptide essential for secretion^23^. Nonetheless, it is conceivable that this subset may be secreted via a non-classical secretion pathway, functioning as an extracellular protease. Indeed, differentiated keratinocytes produce various essential elements that regulate cellular behaviors in both the cytoplasmic and extracellular environments^13,30,31^.

We scrutinized the behaviors of HaCaT keratinocytes, as these cells, in contrast to NHEK cells, express a considerable amount of Pro-FLG with minimal mesotrypsin in conventional culture media. Although exogenous active mesotrypsin seems to undergo rapid degradation in HaCaT cells, prior research has documented the successful production of mesotrypsin in NHEK cells^14,25^. This observation suggested that HaCaT cells may have lost crucial elements for Pro-FLG processing, and this study identifies mesotrypsin as a strong candidate.

In the upper-layer epidermis, active mesotrypsin is derived from its precursor mesotrypsinogen through processing by several proteases, including Cathepsin B, Caspase14, and Enterokinase^23–25^. Alternatively, we explored an inducible expression system for the active form of mesotrypsin, given the difficulty in precisely controlling its functional expression in HaCaT cells. While the intact form of exogenous active mesotrypsin is swiftly degraded by the proteasome, fusion with the Venus protein significantly impedes protein elimination. Although mesotrypsin can undergo chymotrypsin C-triggered self-degradation, as observed in other tissues^46^, a subpopulation of mesotrypsin in the fusion protein evades the protein elimination system, appearing to induce several characteristics in HaCaT cells mirroring epidermal upper-layer keratinocytes.

Despite mesotrypsin inducing a flattened morphology in HaCaT cells, no morphological changes were observed in Cos7 cells. These findings suggested that the mesotrypsin targets responsible for this phenomenon are epidermal cell-specific proteins. The cuboidal-squamous transition in epithelial cells is known to be caused by mechanical properties^47,48^, and keratins act as the primary regulators in keratinocytes^7,49^. Given that FLG functions by bundling keratin and promoting epidermal flattening^2,16^, it is conceivable that this effect was induced, at least in part, through the temporal regulation of keratin dynamics caused by the Pro-FLG processing. While a preceding inquiry observed the existence of multinucleated cells in giant HaCaT cells^26^, mesotrypsin did not induce such cellular phenomena. However, notably, the expression level of mesotrypsin and phenotypic manifestation did not entirely correlate (data not shown), likely due to the ongoing proteasomal degradation of exogenous mesotrypsin.

In addition to exhibiting a flattened morphology, keratinocytes in the granular layer distinctively form TJs at the cell–cell boundary, supported by stable AJs^50^. The expression of mesotrypsin upregulated the major AJ compartment E-cadherin to enhance cadherin-mediated intercellular adhesive strength, followed by accelerated membrane translocation of the TJ scaffold protein ZO-1. While the regulation of the expression profile of these intercellular junctional components is influenced by cytoskeletal rearrangement involving F-actin^51^ and keratin intermediate filaments^52^, both of which are impacted by mesotrypsin, further experiments are requisite to elucidate the molecular mechanism facilitating tight junction formation under the influence of mesotrypsin.

Another noteworthy effect of mesotrypsin is its ability to induce G1 growth arrest in HaCaT cells. In the epidermis of the skin, mitotic cells are predominantly confined to basal layer keratinocytes, and nascent daughter cells promptly cease the cell cycle upon outward extrusion^53^, where mesotrypsin expression has not yet manifested. This observation implies that the mesotrypsin-induced growth arrest in HaCaT cells does not correspond to the cell cycle exit in the normal epidermis, highlighting the necessity for meticulous investigation to delineate the precise role of mesotrypsin on the keratinocyte proliferation. Nonetheless, considering the presence of proliferative keratinocytes often observed in the upper epidermal layers of precancerous lesions, such as actinic keratosis^54^, the effects elucidated in this study could support the proposition that mesotrypsin possesses an intrinsic capacity to act as a backup regulator for preventing re-entry into the growth cell cycle, even within normal keratinocytes. On the other hand, HaCaT cells expressing mesotrypsin undergo the initiation of cell senescence, as evidenced by the significant reduction in laminB1 expression, a phenomenon also observable in the upper layers of the epidermis even in young skin^5^.

Keratinocytes exhibit a distinctive quality wherein the same cellular entities undergo significant alterations in their growth profile, cellular morphology, and intercellular junctions during stratification. Despite the prevailing notion in most prior investigations that these physical properties are intricately linked to keratinocyte differentiation, our study illustrates the induction of cells that exhibit physical characteristics associated with upper-layer keratinocytes but are not undergoing differentiation. This finding suggested the potential omission of intricate epidermal differentiation in the examination of keratinocyte cell behaviors, particularly concerning our long-term-maintained HaCaT cells in the conventional high-calcium medium. Nevertheless, this cellular system may afford notable advantages in elucidating the crucial regulators of keratinocyte morphological behaviors, which are synergistically facilitated by easily manipulable gene transfection.

## Methods

### Cells

Normal human epidermal keratinocytes (NHEKs) (FC-0025, Lifeline Cell Technology, Frederick, USA; purchased from Kurabo, Osaka, Japan) were cultured in Humedia-KG2 medium supplemented with growth factors (Kurabo). The cells were exposed to 2 mM CaCl_2_ for 2 days to induce differentiation. The human keratinocyte cell line HaCaT (gift from Dr. Manabe) has been maintained for more than ten years in DMEM/Ham’s F12 (DH) medium (Fujifilm-Wako, Osaka, Japan) supplemented with 10% heat-inactivated fetal bovine serum (FBS) (CELLect, MP Biomedicals, Illkirch, France), 50 U/mL penicillin, and 50 μg/mL streptomycin (Meiji Seika Pharma, Osaka, Japan) (DH10). These cells, their transgenic derivatives, and Cos7 cells were cultured in DH10 medium. For certain cultures, the cells were treated with 5 μM MG132 (Abcam, Cambridge, UK) for 4 or 7 h. For induction of tight junction (TJ) formation, cells were treated with 10 mM CaCl_2_ and 40 μM the c-Jun N-terminal kinase (JNK) inhibitor SP600125 (Sigma‒Aldrich, St. Louis, USA) for 24 h.

### Generation of HaCaT cells with active mesotrypsin

To construct the tetracycline/doxycycline-inducible expression vector for mesotrypsin, cDNA encoding processed active mesotrypsin (from Ile-81 to Ser-304, accession number; P35030) was amplified by PCR using a plasmid (RIKEN, IRAK0 62C01) as the template. Subsequently, the cDNA encoding T7-tag (MYSMQLASCVTLTLVLLVN) was fused at the C-terminal sequence, after which the resulting cDNA was inserted into the Not I site in PBtet0606 vector^11,35^ to generate PBtet0606-mesotrypsin. For construction of the inducible expression vector for the Venus-mesotrypsin fusion protein, cDNA encoding the entire Venus protein (excluding the stop codon) was sequentially fused with synthetic double-strand DNA encoding a linker sequence (GGGGSGGSGGGSTQGEL), followed by the TEV protease recognition sequence (ENLYFQG) and the cDNA of the processed active mesotrypsin (from Ile-81 to Ser-304). Venus, a modified form of GFP (accession number; P42212), was utilized for facile detection of the transgene in living cells. The resulting DNA construct was subsequently inserted into the PBtet0606 vector, yielding PBtet0606-Venus-mesotrypsin. As a control, the plasmid PBtet0606-Venus lacking the mesotrypsin cDNA sequence from PBtet0606-Venus-mesotrypsin was also prepared. To establish HaCaT cells with dox-inducible expression of mesotrypsin or Venus-mesotrypsin, cells were transfected with PBtet0606-mesotrypsin or PBtet0606-Venus-mesotrypsin in conjunction with PB-CA-rtTA-hygro^11^ and CAG-PBase (Addgene, Watertown, USA) using the electroporator CUY21Pro-Vitro (Nepa gene, Chiba, Japan). Cells displaying resistance to both 100 μg/mL neomycin and 50 μg/mL hygromycin were assessed for inducible transgene expression upon treatment with 5 μg/ml dox and subsequently mixed for subsequent experiments. The transient expression of the transgenes in Cos7 cells was evaluated using Lipofectamine 2000 (Invitrogen-Thermo Fisher).

### Preparation of recombinant GFP-mesotrypsin protein

To construct a bacterial expression plasmid for the production of recombinant GFP-mesotrypsin protein, cDNA encoding linker-mesotrypsin was ligated to the C-terminal sequence of 6 × Histidine tagged-GFP and subsequently inserted into the pet 3a plasmid (Sigma‒Aldrich), yielding pet 3a-GFP-mesotrypsin. For control purposes, an analogous plasmid devoid of mesotrypsin cDNA was also generated (pet3a-GFP). The bacterial strain BL21 was transformed with either of these plasmids, cultured at 32 °C, and induced with isopropyl β-d-1-thiogalactopyranoside (IPTG) at 32 °C for 2 h. The resultant recombinant protein within the bacterial cells was purified utilizing NI–NTA columns (Qiagen, Hilden, Germany) and subsequently dialyzed against PBS. Both recombinant proteins yielded more than 1 mg/mL. The purified proteins were bound to NI–NTA beads (Qiagen) again and incubated with HaCaT cell lysate extracted in TBS containing 1% Triton X-100 for 1 h. The cell lysate was then analyzed for Pro-FLG.

### Immunodetection

Western blotting was performed following established protocols, employing ECL reagent (Invitrogen-Thermo Fisher) for detection and LAS-500 (GE HealthCare, Chicago, USA) for analysis. When analyzing multiple proteins in a blot, protein-transferred membranes were cut into sections containing the target protein bands before incubation with antibodies (Thus, we cannot present full membranes for some samples; see Supplementary Fig. S4). Immunocytochemistry was performed as follows: Cells were cultured on a chamber slide (SPL Life Science, Gyeonggi-do, Korea) precoated with collagen and fixed with 4% paraformaldehyde in Tris-buffered saline (TBS) for 10 min. Subsequently, the cells were permeabilized with 0.1% Triton X-100 for 10 min, incubated with 4% skim milk in TBS (STBS) for 1 h, and incubated with primary antibodies in STBS for 2 h. Following TBS washing, the cells were treated with labeled secondary antibodies and counterstained with Hoechst 33258 (Dojindo, Kumamoto, Japan). Analysis of the samples was conducted using a TCS SPE system (Leica, Wetzlar, Germany). The primary antibodies utilized in this study included antibodies against β-actin (Sigma‒Aldrich), TGase-1 (Proteintech, Rosemont, USA), E-cadherin (ECCD2, a gift from Dr. Takeichi), LaminA/C (Cell Signaling, Danvers, USA), Lamin B1 (Abcam, Cambridge, UK), Rb, phosphorylated Rb (Cell Signaling Technology, Danvers, USA), ZO-1 (a gift from Dr. Nagafuchi), T7-tag (MBL, Tokyo, Japan), Ki67 (Abcam), Pro-FLG^12^, and GFP/Venus (culture supernatant of JFP-J1 cells, Riken Cell Bank RCB2309). Secondary antibodies conjugated to Alexa488, Cy3, or horseradish peroxidase (HRP) were obtained from Sigma‒Aldrich and Merck-Millipore (Darmstadt, Germany). To quantify the accumulation of ZO-1 or E-cadherin, cells were stained for these molecules and photographed using a TCS SPE system (Leica, Wetzlar, Germany). A closed line surrounding each cell was then traced, and the total signal intensity was determined using the ImageJ system^55^.

### Cell proliferation assay

In addition to analyzing Ki67-positive cells, we further evaluated the cell growth potential of the cells utilizing the Alamar Blue reagent. The cells were cultured in 96-well plates and exposed to 10% Alamar Blue (Invitrogen-Thermo Fisher) in DH10 medium. After incubating at 37 °C for 1 h, the absorbances at 570 and 600 nm were quantified using a Multiskan FC microplate reader (Invitrogen-Thermo Fisher), and the ratio of the change to the control was subsequently calculated.

### Evaluation of cadherin-mediated cell adhesion strength

To assess the robustness of cadherin-mediated intercellular adhesion, a cell aggregation assay was conducted utilizing cells expressing only cadherins as intercellular adhesion molecules, as previously described^56^. The cells were treated with 0.01% trypsin in HEPES-buffered saline containing 2 mM CaCl2, which effectively eliminated most adhesion molecules while preserving cadherins^56^ (Supplementary Fig. S3). Subsequently, the cells were thoroughly dissociated through vigorous pipetting, centrifuged, and reconstituted in a DH10 medium. After rotation at 100 rpm for 60 min, the size of the newly formed cell aggregates, which reflects the adhesive strength of the cadherin, was quantified.

### Quantitative real-time PCR (qRT‒PCR)

Total RNA was extracted utilizing a Total RNA Extraction Miniprep System (VIOGENE, Taipei, Taiwan) and reverse-transcribed with ReverTra Ace (TOYOBO Shiga, Japan). Quantitative real-time PCR (qRT–PCR) was performed using Fast Start Essential DNA Green Master on a Thermal Cycler Dice system (Takara). *PRSS3* (*Mesotrypsin*), *CDH1* (*E-cadherin*), *MYC* (*c-Myc)*, *CDKN1A* (*P21)*, *IVL* (*Involucrin*), *LOR* (*Loricrin*), *TGM1* (*TGase-1*), and *GAPDH* were amplified with the primer pairs listed in Table 1. The expression of each mRNA was normalized to that of *GAPDH*.

**Table 1:** Primer sets for qRT‒PCR.

Gene accession number	Forward primer	Reverse primer
*GAPDH* NM_002046	5ʹ-GGTGTGAACCATGAGAAGTATGA-3ʹ	5ʹ-GAGTCCTTCCACGATACCAAAG-3ʹ
*PRSS3* NM_007343.4 *(Mesotrypsin)*	5ʹ-CTTCCTTGAGGGAGGCAAGG-3ʹ	5ʹ-CTCCAGGCCTGTTCTTCCAG-3ʹ
*T7*	5ʹ-CTTCCTTGAGGGAGGCAAGG-3ʹ	5ʹ-ACCCATTTGCTGTCCACC-3ʹ
*CDH1* NM_004360 *(E-cadherin)*	5ʹ-GGTGCTCTTCCAGGAACCTC-3ʹ	5ʹ-GTTCCATAAATGTGTCTGGCTCC-3ʹ
*TGM1* NM_000359 *(TGase1)*	5ʹ-GGGAACTGGTCTGGTGATTA-3ʹ	5ʹ-AATATCCCGTGCGTAGGTAG-3ʹ
*IVL* NM_005547 *(Involucrin)*	5ʹ-AAAGCAGAAAACCCAGAGCA-3ʹ	5ʹ-CTCTAGGTGCTTCAGGTGCC-3ʹ
*LOR* NM_000427 *(Loricrin)*	5ʹ-CATGATGCTACCCGAGGTTT-3ʹ	5ʹ-ACTGGGGTTGGGAGGTAGTT-3ʹ
*MYC* NM_002467 *(c-Myc)*	5ʹ-TAACGTTGAGGGGCATCG-3ʹ	5ʹ-GCTGCTTAGACGCTGGAT TT-3ʹ
*CDKN1A* NM_078467 *(p21)*	5ʹ-TCGCTCAGGGGAGCAGGCTGA-3ʹ	5ʹ-TGCGCTTGGAGTGATAGA AA-3ʹ

### Statistical analyses

The data are presented as the mean ± SD from a minimum of three independent experiments. Student’s t-test was used to determine the p values, with values less than 0.05 considered to indicate statistical significance. In cases involving three or more samples, multiple testing was conducted using one-way ANOVA and the Tukey test.

### Supplementary Information

Supplementary Figure S1.

Supplementary Figure S2.

Supplementary Figure S3.

Supplementary Figure S4.

Supplementary Figure S4.

Supplementary Figure S4.

Supplementary Figure S4.

Supplementary Figure S4.

Supplementary Figure S4.

Supplementary Figure S4.

Supplementary Figure S4.

Supplementary Figure S4.

## Data Availability

The datasets are included in Supplementary Figs. S2–S4. Also, all materials used in this study and the datasets are available from the corresponding author upon reasonable request.
