# Supplementary figures and images for: PRSS3/mesotrypsin as a putative regulator of the biophysical characteristics of epidermal keratinocytes in superficial layers
Source: Sci Rep. 2024 May 29;14:12383. doi: 10.1038/s41598-024-63271-w (PMC11137022; doi:10.1038/s41598-024-63271-w)

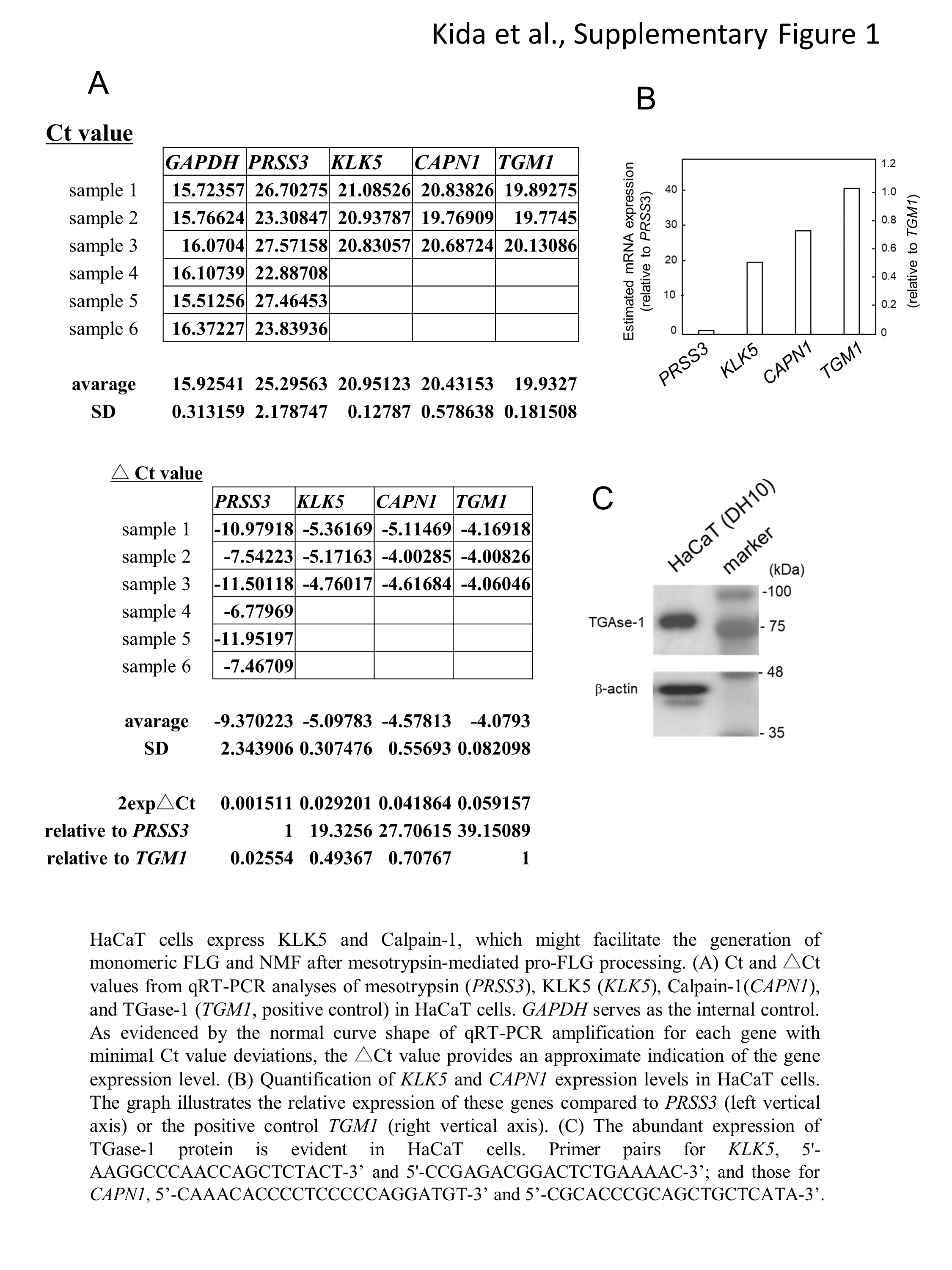

Supplement: Supplementary file 1 — Supplementary Figure S1. [file 41598_2024_63271_MOESM1_ESM.tif]

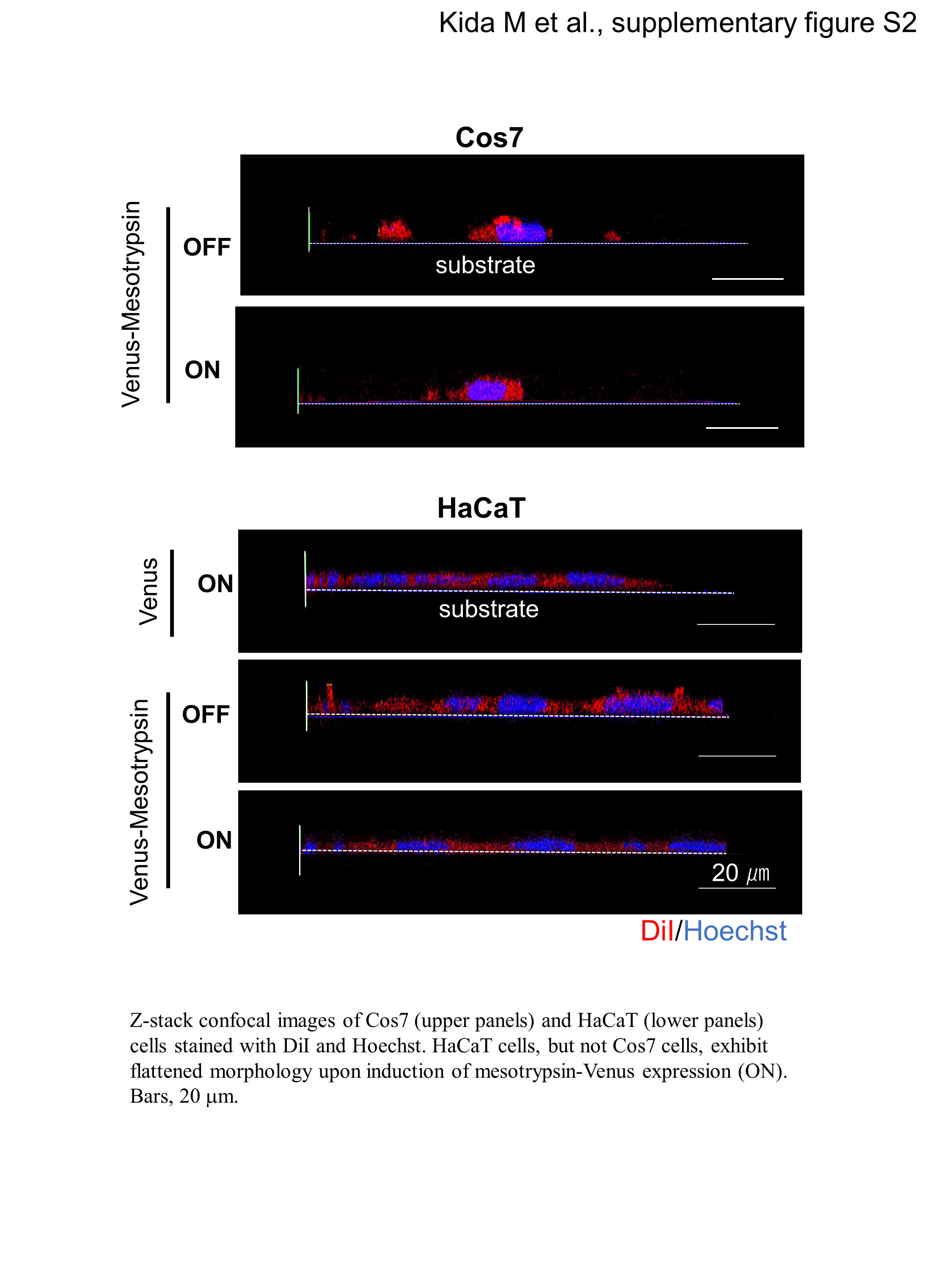

Supplement: Supplementary file 2 — Supplementary Figure S2. [file 41598_2024_63271_MOESM2_ESM.tif]

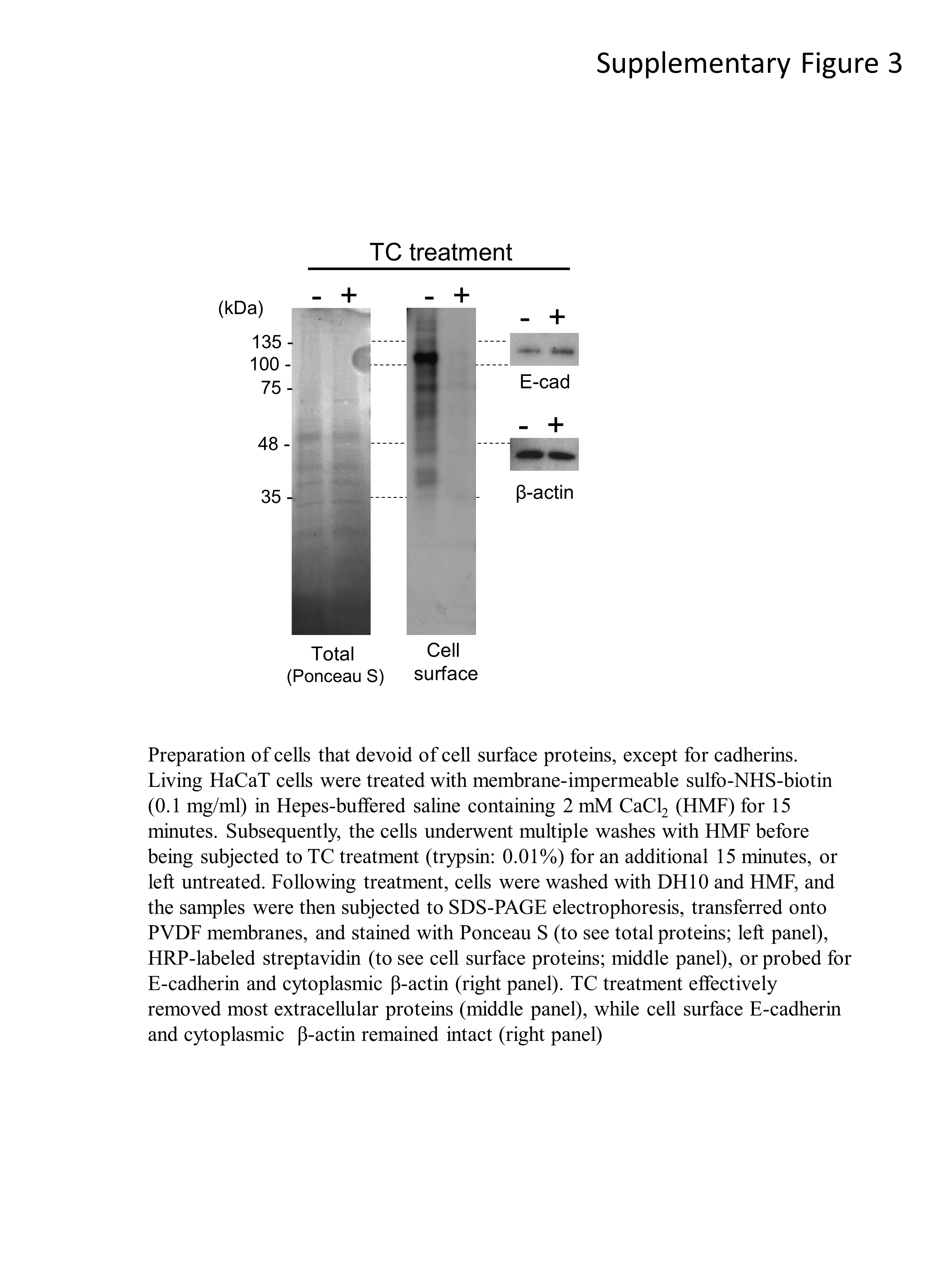

Supplement: Supplementary file 3 — Supplementary Figure S3. [file 41598_2024_63271_MOESM3_ESM.tif]

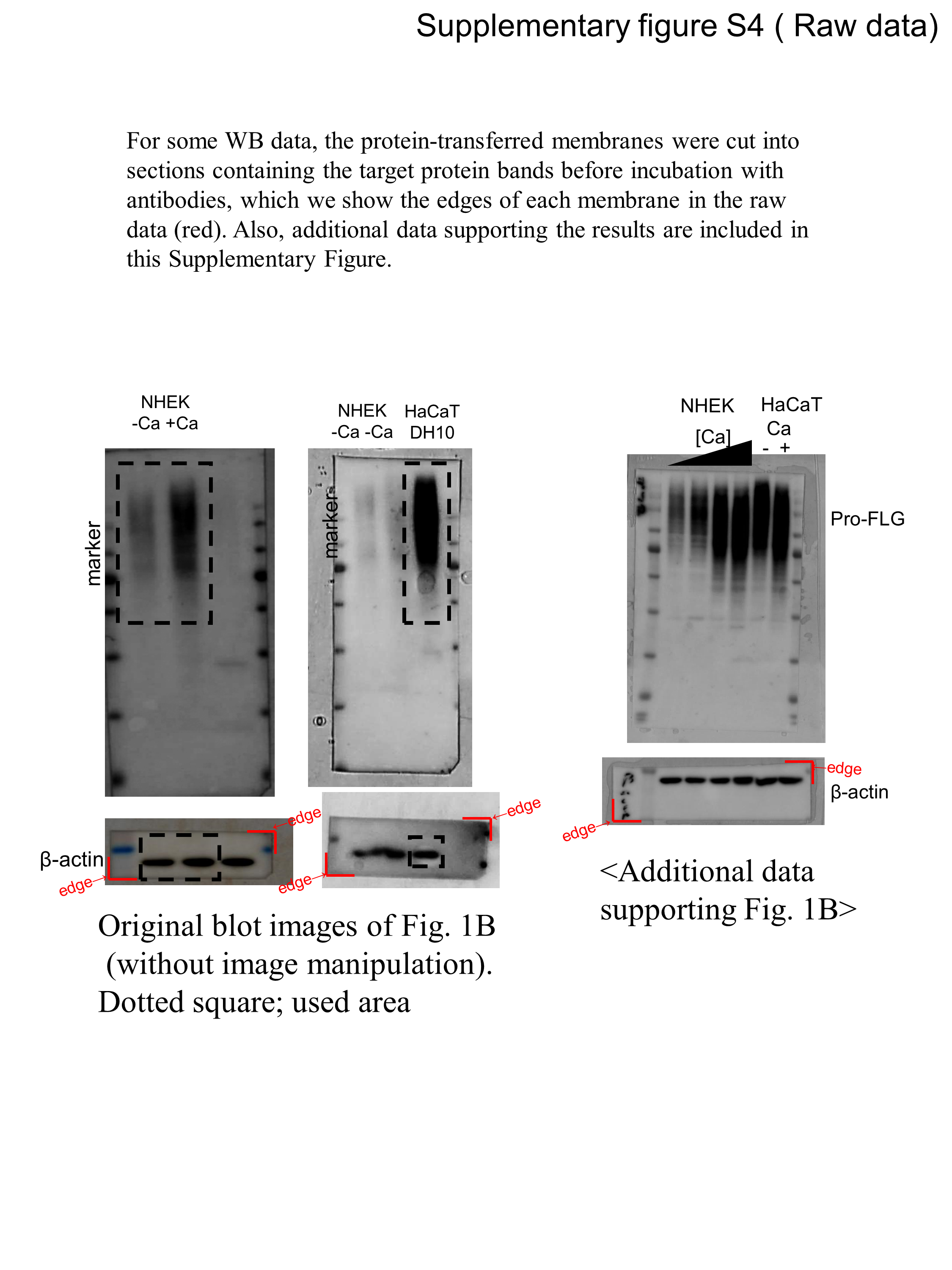

Supplement: Supplementary file 4 — Supplementary Figure S4. [file 41598_2024_63271_MOESM4_ESM.tif]

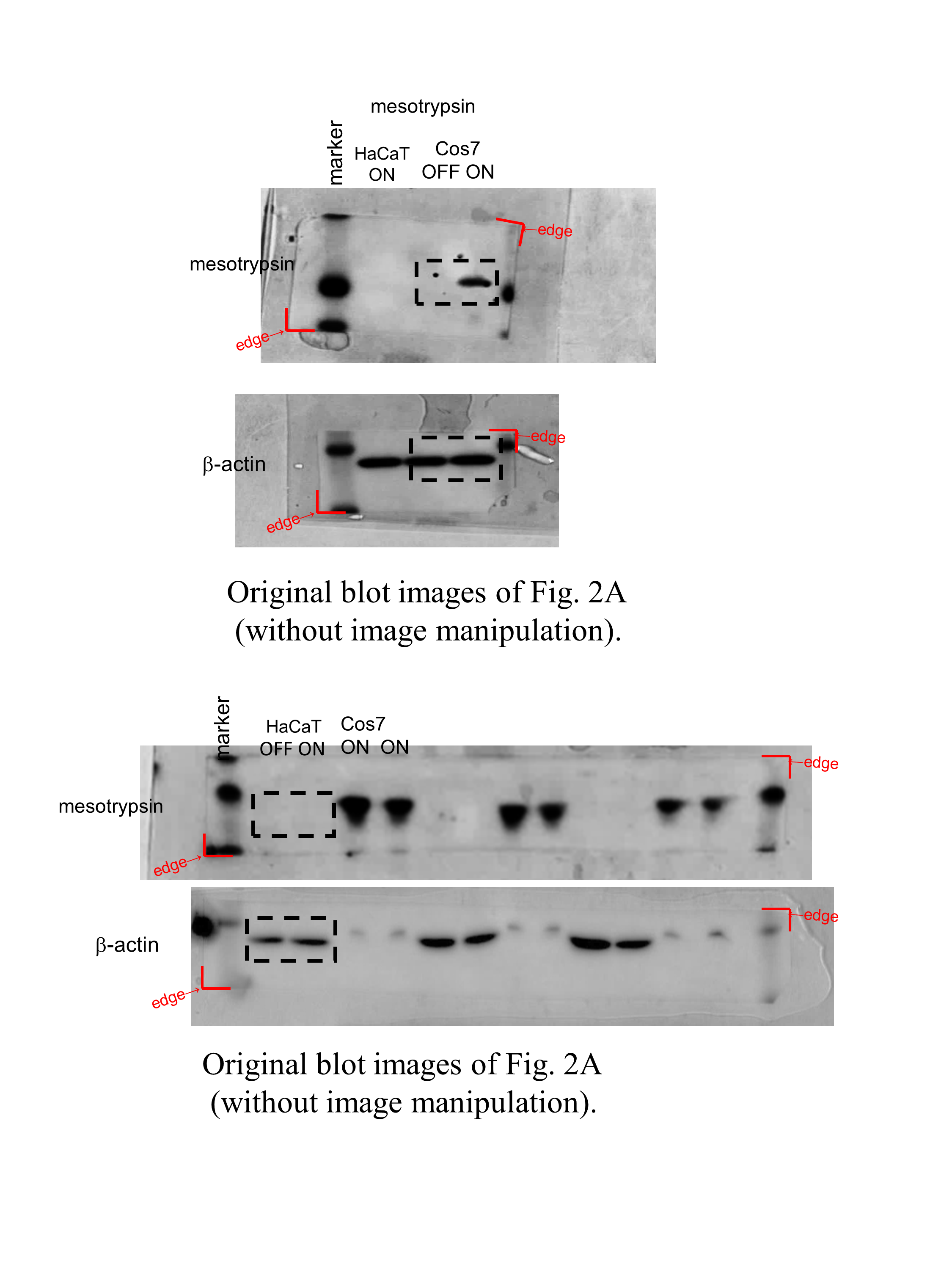

Supplement: Supplementary file 5 — Supplementary Figure S4. [file 41598_2024_63271_MOESM5_ESM.tif]

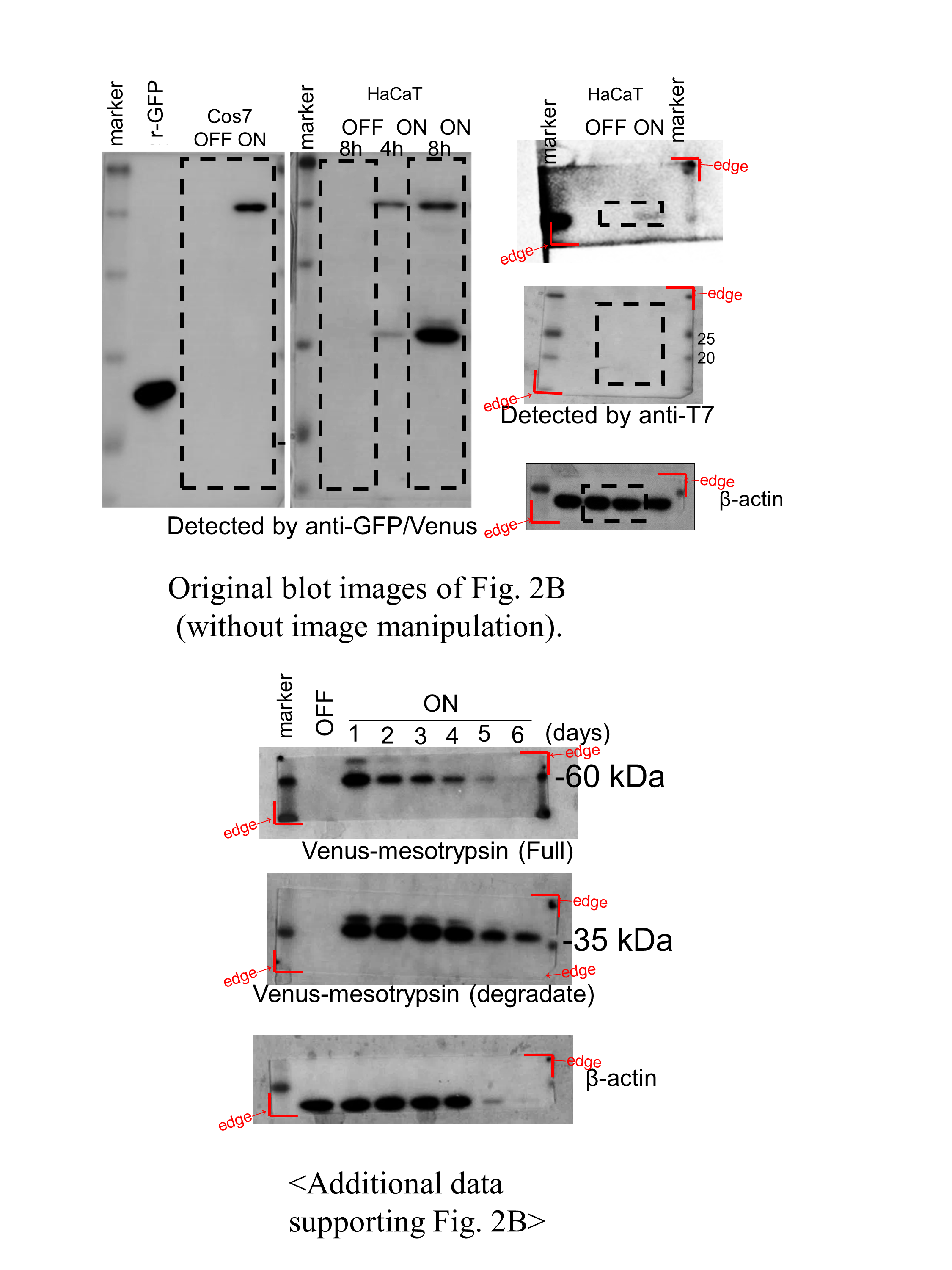

Supplement: Supplementary file 6 — Supplementary Figure S4. [file 41598_2024_63271_MOESM6_ESM.tif]

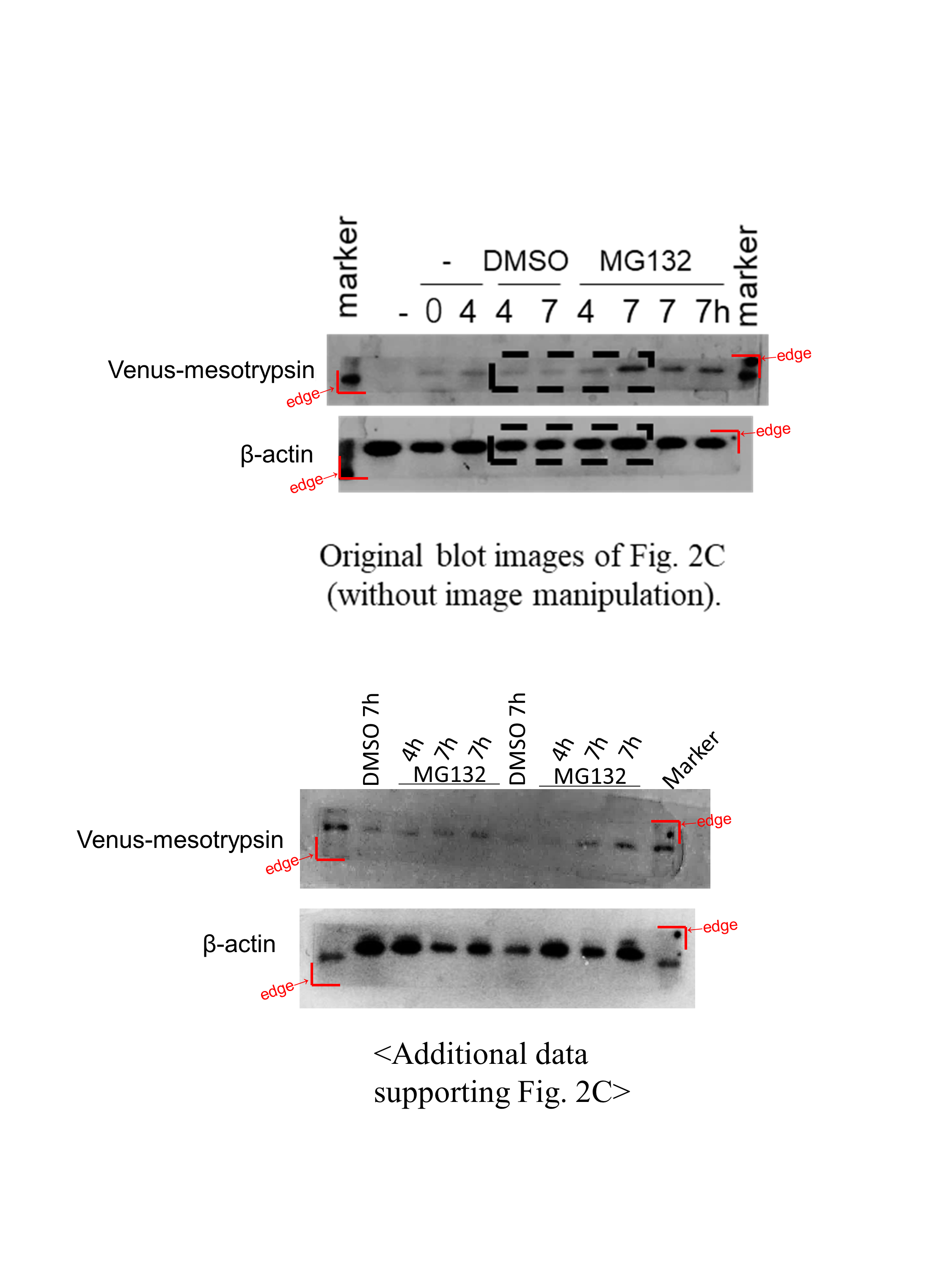

Supplement: Supplementary file 7 — Supplementary Figure S4. [file 41598_2024_63271_MOESM7_ESM.tif]

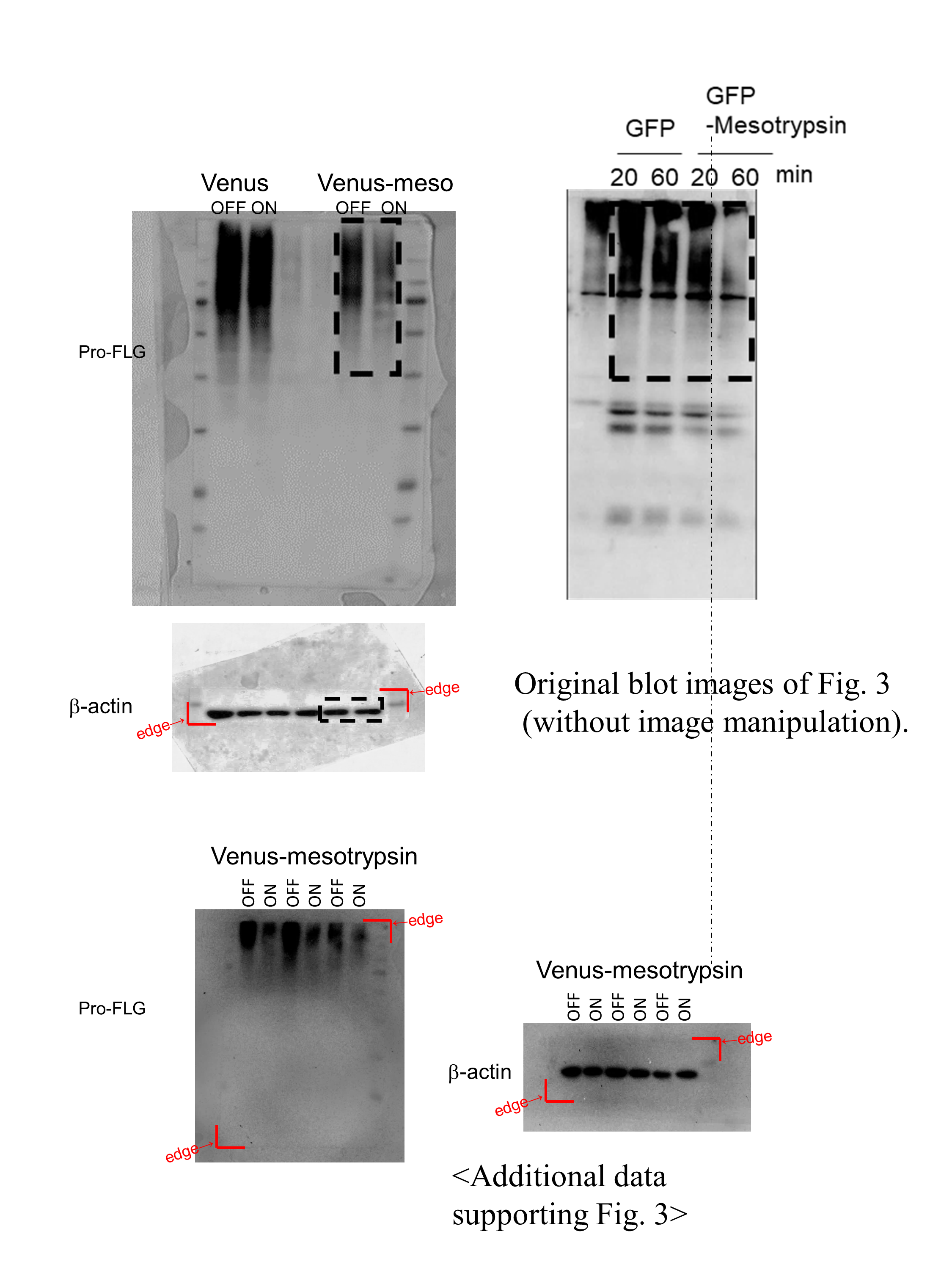

Supplement: Supplementary file 8 — Supplementary Figure S4. [file 41598_2024_63271_MOESM8_ESM.tif]

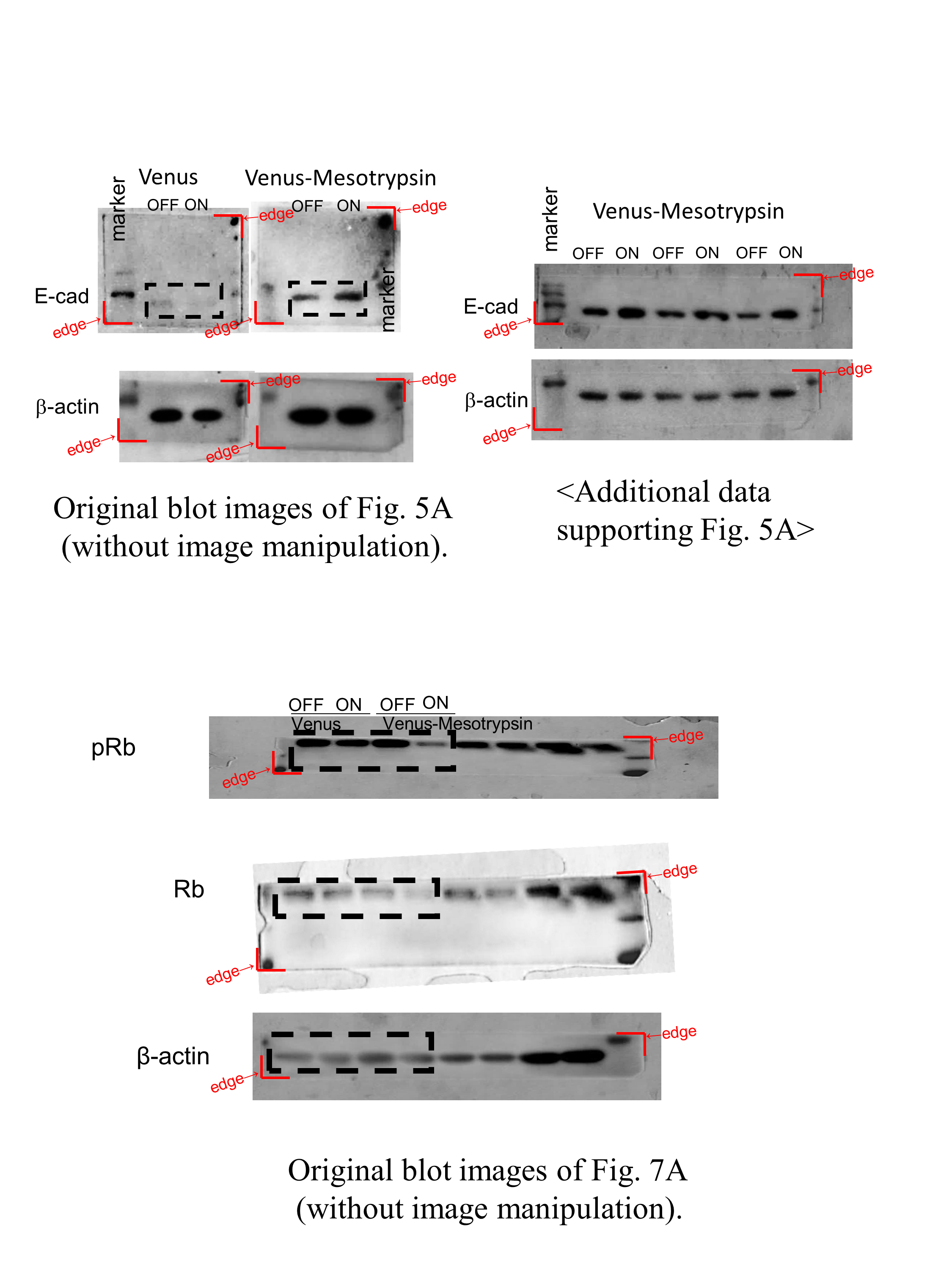

Supplement: Supplementary file 9 — Supplementary Figure S4. [file 41598_2024_63271_MOESM9_ESM.tif]

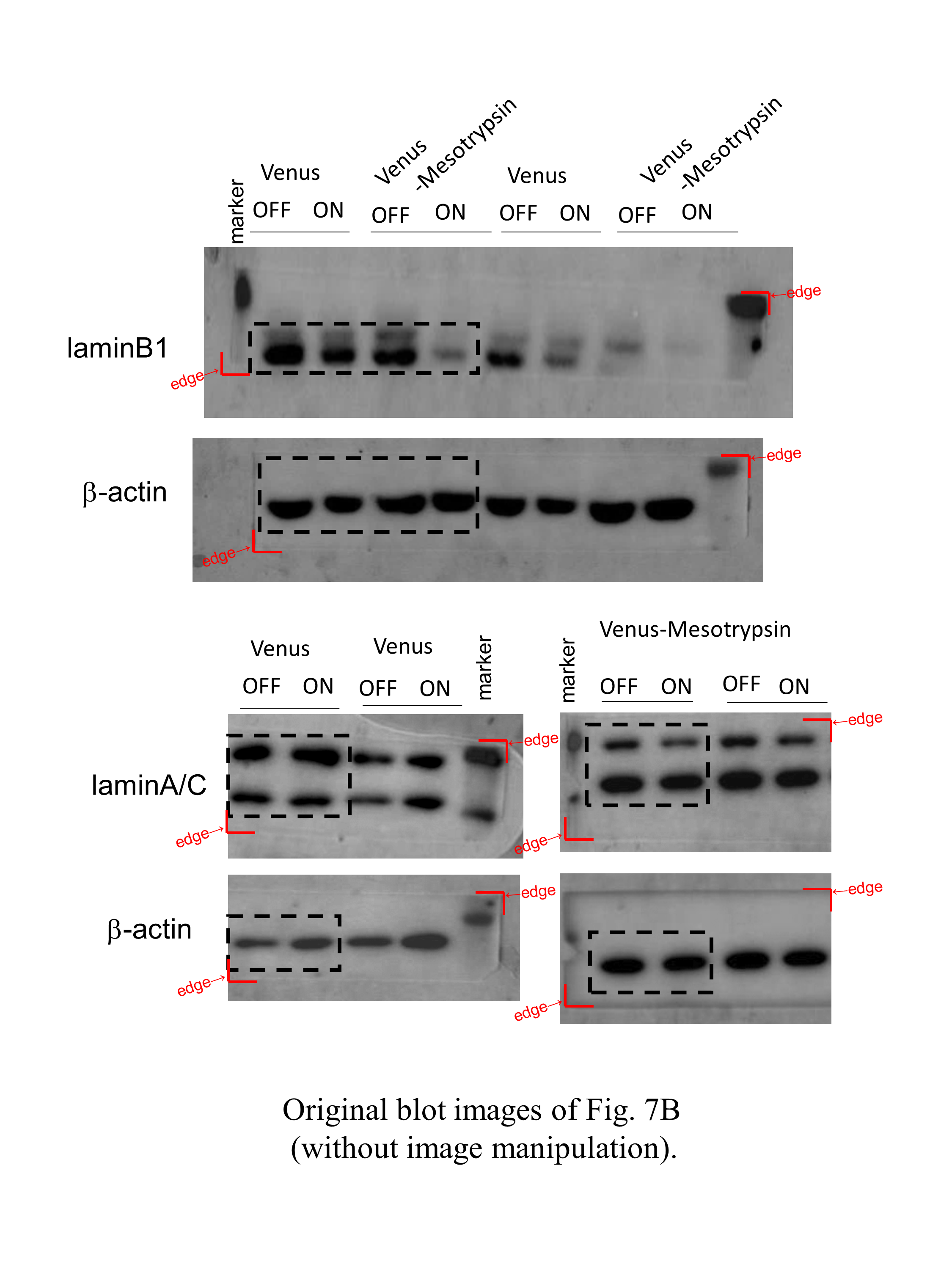

Supplement: Supplementary file 10 — Supplementary Figure S4. [file 41598_2024_63271_MOESM10_ESM.tif]

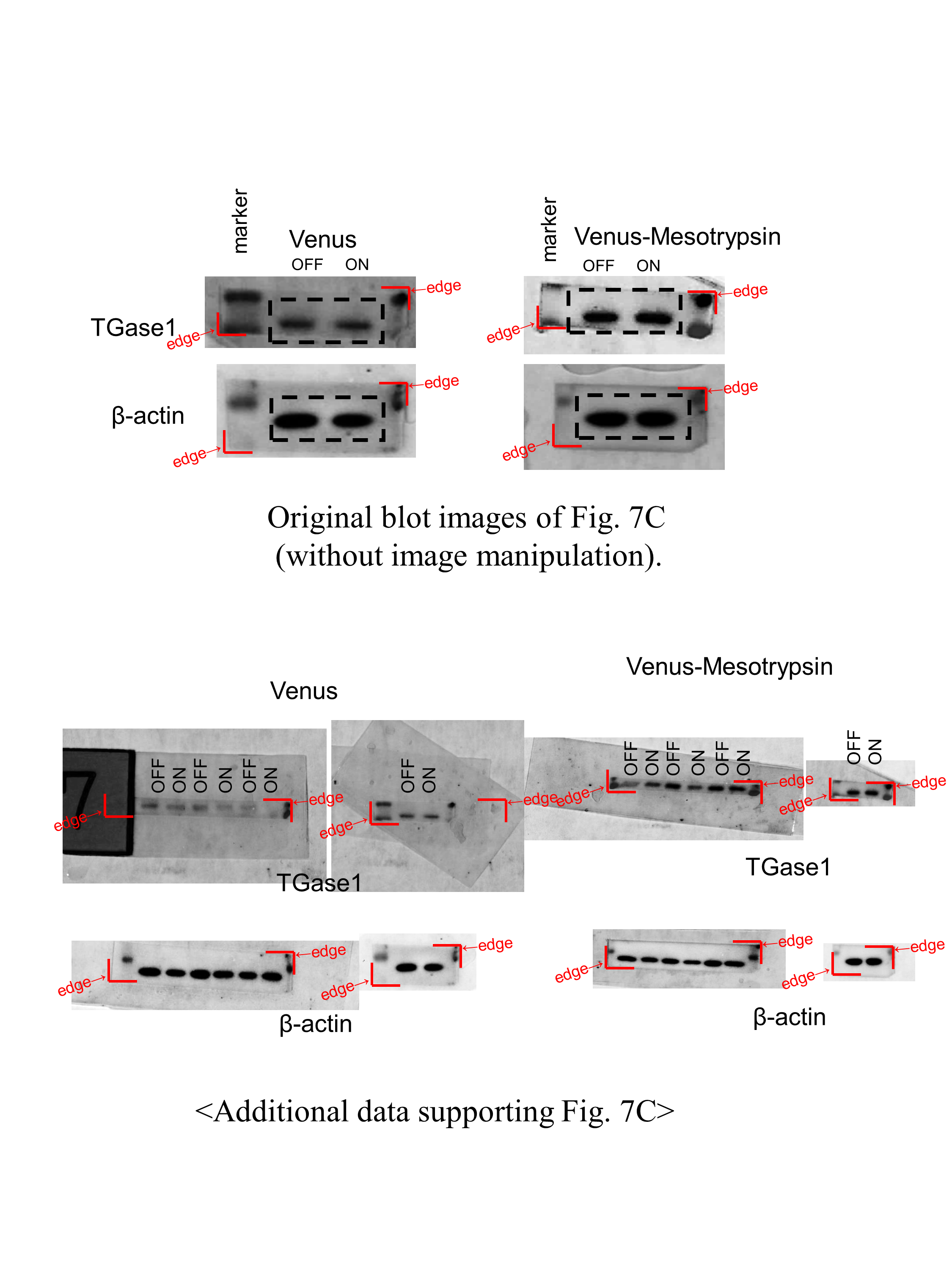

Supplement: Supplementary file 11 — Supplementary Figure S4. [file 41598_2024_63271_MOESM11_ESM.tif]

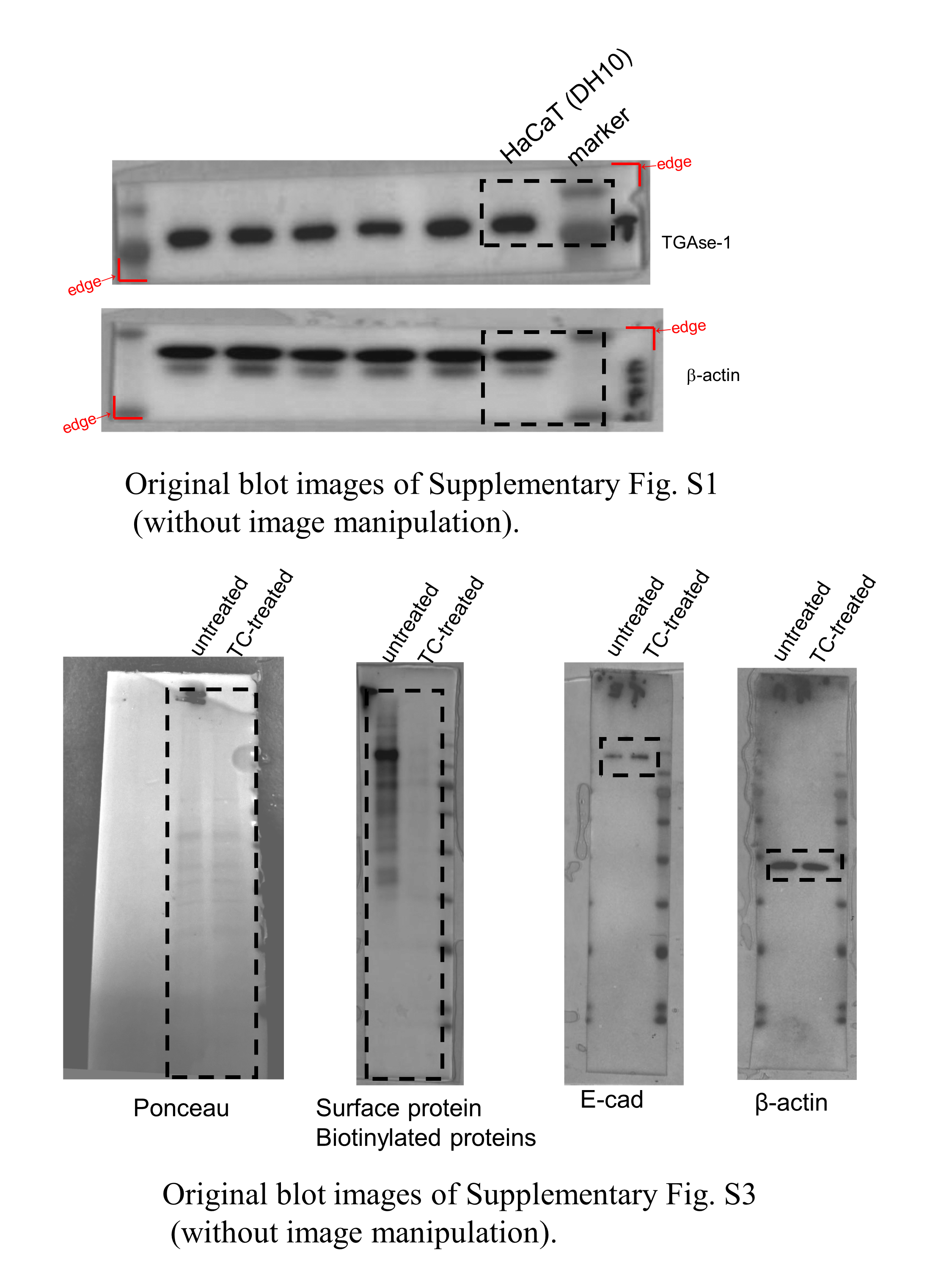

Supplement: Supplementary file 12 — Supplementary Figure S4. [file 41598_2024_63271_MOESM12_ESM.tif]
